# Global population structure, genomic diversity and carbohydrate fermentation characteristics of clonal complex 119 (CC119), an understudied Shiga toxin-producing *E. coli* (STEC) lineage including O165:H25 and O172:H25

**DOI:** 10.1099/mgen.0.000959

**Published:** 2023-03-23

**Authors:** Keiji Nakamura, Kazuko Seto, Kenichi Lee, Tadasuke Ooka, Yasuhiro Gotoh, Itsuki Taniguchi, Yoshitoshi Ogura, Jacques Georges Mainil, Denis Piérard, Tetsuya Harada, Yoshiki Etoh, Saori Ueda, Mitsuhiro Hamasaki, Junko Isobe, Keiko Kimata, Hiroshi Narimatsu, Jun Yatsuyanagi, Makoto Ohnishi, Sunao Iyoda, Tetsuya Hayashi

**Affiliations:** ^1^​ Graduate School of Medical Sciences, Kyushu University, Fukuoka, Japan; ^2^​ Osaka Institute of Public Health, Osaka, Japan; ^3^​ National Institute of Infectious Diseases, Tokyo, Japan; ^4^​ Graduate School of Medical and Dental Sciences, Kagoshima University, Kagoshima, Japan; ^5^​ Kurume University School of Medicine, Fukuoka, Japan; ^6^​ Faculty of Veterinary Medicine, University of Liege, Liege, Belgium; ^7^​ Universitair Ziekenhuis Brussel (UZ Brussel), Vrije Universiteit Brussel (VUB), Brussels, Belgium; ^8^​ Fukuoka Institute of Health and Environmental Sciences, Fukuoka, Japan; ^9^​ Toyama Institute of Health, Toyama, Japan; ^10^​ Oita Prefectural Institute of Health and Environment, Oita, Japan; ^11^​ Akita Prefectural Institute of Public Health, Akita, Japan; ^†^​ Present address: Chūbu Regional Public Health Center, Okinawa, Japan

**Keywords:** O165:H25, O172:H25, comparative genomics, carbohydrate fermentation, phylogenetic analysis, Shiga toxin-producing *Escherichia coli*

## Abstract

Among Shiga toxin (Stx)-producing *Escherichia coli* (STEC) strains of various serotypes, O157:H7 and five major non-O157 STEC (O26:H11, O111:H8, O103:H2, O121:H19 and O145:H28) can be selectively isolated by using tellurite-containing media. While human infections by O165:H25 STEC strains have been reported worldwide, their detection and isolation are not easy, as they are not resistant to tellurite. Systematic whole-genome sequencing (WGS) analyses have not yet been conducted. Here, we defined O165:H25 strains and their close relatives, including O172:H25 strains, as clonal complex 119 (CC119) and performed a global WGS analysis of the major lineage of CC119, called CC119 *sensu stricto* (CC119ss), by using 202 CC119ss strains, including 90 strains sequenced in this study. Detailed comparisons of 13 closed genomes, including 7 obtained in this study, and systematic analyses of Stx phage genomes in 50 strains covering the entire CC119ss lineage, were also conducted. These analyses revealed that the Stx2a phage, the locus of enterocyte effacement (LEE) encoding a type III secretion system (T3SS), many prophages encoding T3SS effectors, and the virulence plasmid were acquired by the common ancestor of CC119ss and have been stably maintained in this lineage, while unusual exchanges of Stx1a and Stx2c phages were found at a single integration site. Although the genome sequences of Stx2a phages were highly conserved, CC119ss strains exhibited notable variation in Stx2 production levels. Further analyses revealed the lack of SpLE1-like elements carrying the tellurite resistance genes in CC119ss and defects in rhamnose, sucrose, salicin and dulcitol fermentation. The genetic backgrounds underlying these defects were also clarified.

## Data Summary

The closed genomes of 7 strains and the Illumina read sequences of 73 O165:H25 and 10 O172:H25 strains obtained in this study have been deposited in DDBJ/EMBL/GenBank under BioProject accession numbers starting from PRJDB8147 (https://www.ncbi.nlm.nih.gov/bioproject, see Table S1, available in the online version of this article, for the accession numbers of each strain). All Stx phage genome sequences determined in this study have been deposited in the DDBJ/EMBL/GenBank databases under the accession numbers listed in Table S2.

**Impact Statement**

Tellurite resistance is a useful biochemical marker to isolate major Shiga toxin (Stx)-producing *Escherichia coli* (STEC) strains such as O157:H7. Here, we present the results of a series of whole-genome sequencing (WGS)-based analyses of a global set of strains belonging to clonal complex 119 (CC119), including the O165:H25 and O172:H25 strains, which are apparently understudied due to their lack of tellurite resistance. Our analyses revealed the acquisition history and conservation of major STEC virulence factors and mobile genetic elements encoding them in the major lineage of CC119 and the lack of SpLE1-like elements carrying the tellurite resistance genes. Defects in the fermentation of four carbohydrates and the genetic backgrounds underlying these defects were also identified, including the defect in rhamnose fermentation, which is atypical among *E. coli* strains and thus will be useful to detect and identify strains belonging to this understudied STEC lineage.

## Introduction

Shiga toxin (Stx)-producing *Escherichia coli* (STEC) are foodborne pathogens causing diarrhoea and haemorrhagic colitis (HC) with life-threatening complications, such as haemolytic uraemic syndrome (HUS). The major virulence factors of STEC are Stxs encoded by prophages (PPs). Stxs are classified into Stx1 and Stx2, and both types include multiple variants (Stx1a, Stx1c and Stx1d; Stx2a–Stx2g) [1]. Typical STEC possess the locus of enterocyte effacement (LEE) element encoding a type III secretion system (T3SS) that secretes more than 30 LEE- or PP-encoded effectors [2–4]. In addition, most major STEC strains carry a large virulence plasmid, and enterohaemolysin and several other potential virulence factors are encoded therein [4, 5].

Among various STEC serotypes, O157:H7 and five non-O157 serotypes (O26:H11, O111:H11, O103:H2 O121:H19 and O145:H28) frequently cause outbreaks and sporadic cases of infection worldwide [6, 7]. Strains having these serotypes evolved independently into typical STEC possessing a similar set of virulence factors via the acquisition of Stx-converting phages (Stx phages), LEE, T3SS effector-encoded PPs and the virulence plasmid [4, 8, 9]. Another important mobile genetic element (MGE) common to the major STEC serotypes is an integrative element (IE) called SpLE1 [10] or a SpLE1-like element. This element contains genes encoding an integrase and the insertion sequence (IS) excision enhancer (IEE) [11], a urease gene cluster and *ter* genes responsible for tellurite resistance [12]. As tellurite inhibits the growth of most *E. coli* strains in normal intestinal flora [13], STEC are selectively isolated on potassium tellurite-containing media, such as cefixime, tellurite–sorbitol–MacConkey (CT-SMAC) agar [14]. Therefore, the use of tellurite is one of the most effective methods to detect and isolate STEC strains. However, some clinically relevant STEC serotypes are sensitive to tellurite, including O91 serogroups (O91:H8, O91:H10, O91:H14 and O91:H21), O113:H21 [15–17] and O165:H25 [18].

STEC O165:H25 (or O165:H-) has also been isolated from HC and HUS patients worldwide [18–27]. These isolates possess *stx* (*stx1*, *stx2a* and/or *stx2c*), *eae* (the marker of the LEE) and *ehxA* (the marker of the virulence plasmids of typical STEC) genes [18, 24, 27, 28], suggesting that the virulence potential of this serotype is probably similar to that of the major STEC serotypes. However, due to the inability to grow on tellurite-containing media [18], the detection and isolation of O165:H25 strains are not easy; thus, it is very likely that the prevalence of this serotype is underestimated. In addition, no comprehensive phylogenomic analyses of the O165:H25 lineage have been conducted.

In this study, O165:H25 strains and their close relatives, including O172:H25 strains, were defined as clonal complex 119 (CC119), and a whole-genome sequencing (WGS) analysis of 202 CC119 strains, including 80 O165:H25 and 10 O172:H25 strains sequenced in this study, was performed to reveal the general genomic features, global population structure and genomic diversity of CC119 strains. A detailed comparison of 13 closed genomes, including 7 genomes closed in this study, was also conducted to show the conservation and/or diversification of MGEs in the CC119 population. The variations in Stx phage genomes and Stx2 production levels among CC119 strains were analysed in more detail. Finally, we found defective phenotypes of the CC119 lineage in the fermentation of four carbohydrates and the genetic bases underlying these unique phenotypes.

## Methods

### Bacterial strains

All O165:H25 and O172:H25 strains sequenced in this study were clinical isolates obtained in Japan and Belgium from 1998 to 2021, with the exception of four strains from asymptomatic carriers (*n*=2) and foods (*n*=2) and four strains without clinical information. The sequence types (STs) of these strains were determined by SRST2 [29] or MLST2.0 (https://cge.food.dtu.dk/services/MLST/) based on Achtman’s scheme of multilocus sequence typing (MLST) [30]. To reveal the global population structure of ST119 (the major ST in our strain set) and its close variants, we searched the National Center for Biotechnology Information (NCBI) and EnteroBase databases (final access: 2 February 2022) for the genome sequences of ST119 and its single-locus variants (SLVs) and double-locus variants (DLVs). Sequences without country information and low-quality sequences [>1000 scaffolds/contigs (>300 bp); <99 % completeness or >1 % contamination assessed by CheckM [31]] were excluded. The serotypes of strains were determined by ECTyper [32]. The final strain set analysed in this study comprised 161 ST119 strains and 41 strains belonging to the SLVs or DLVs of ST119 (34 and 7, respectively), as listed in Table S1 (available in the online version of this article).

### Genome sequencing, assembly and annotation

Purification of genomic DNA, preparation of sequencing libraries and Illumina sequencing were performed as previously described [8]. Assembly, scaffolding and gap-closing of Illumina reads were performed using the Platanus_B_v1.3.2 assembler [33], and scaffolds larger than 300 bp were used in this study. The sizes of assembled genomes, sequence coverages and yielded scaffolds ranged from 5015 to 5573 kb (median: 5254 kb), from 46 to 321× (median: 67×), and from 254 to 775 (median: 394) (Table S1), respectively.

Strain JNE072951 was sequenced using Roche 454 GS FLX with a shotgun and an 8 kb mate-pair library, and the obtained reads were assembled by Roche Newbler. Gap-filling was subsequently performed by PCR and Sanger sequencing of PCR products to obtain the closed genome. To determine the complete sequences of six additional strains (PV06-71, JNE102603, JNE110611, PV01-97, JNE072929 and JNE071324), their genomes were sequenced using MinION with R9.4.1 flow cells (Oxford Nanopore Technologies, ONT) for 96 (JNE071324) or 48 h (the other strains). Read data in FASTQ format were generated using MinKnow v1.13.1 and Albacore v2.3.1 (PV06-71 and JNE102603); MinKnow v1.15.1 and Albacore v2.3.1 (JNE110611, PV01-97 and JNE072929); or MinKnow core v3.1.13, Guppy v1.8.7 and qcat v1.1.0 (JNE071324). Long reads obtained were trimmed by porechop (v0.2.2) [34] with the option of trimming 100 bp from the start of reads and filtered by NanoFilt (v2.3.0) [35] with a threshold of over 2 kb at a quality score of >9 (PV01-97) or >10 (the other strains). The filtered long reads were assembled along with the trimmed (PV06-71, JNE110611, PV01-97 and JNE072929) or raw (JNE102603 and JNE071324) Illumina reads of each strain using Unicycler v0.4.6 [36]. As the chromosome of JNE071324 was not circularized, gap filling was subsequently performed by long PCR, which was followed by Illumina sequencing and assembly of PCR products as previously described [9]. The seven genomes closed in this study were annotated using DFAST [37] and then manually curated. PPs, IEs and ISs were identified as previously described [8]. GenomeMatcher (v3.0.4) [38] was used for genome sequence comparison and to display the results.

### SNP detection and phylogenetic analyses

To reconstruct the phylogeny of *E. coli *, we used the genomes of 104 *E. coli* strains representing each of the 104 serotypes that were selected from the closed chromosomal genomes in the NCBI database (Table S3). For this purpose, we first collected all available closed genomes in the database (final access: 20 July 2019). After determining their serotypes by ECTyper (only when serotype information was not available in the database), 1 genome was selected for each of the 104 serotypes found in the closed genome data set. *Escherichia* cryptic clade I strain TW10509 (no. AEKA00000000) was used as an outgroup. The core genes (*n*=2,647; present in all strains) of these strains were identified by Roary [39], and their concatenated sequence alignments were also generated by Roary. Based on the 100 652 SNP sites extracted from the alignment using SNPsites [40], a maximum-likelihood (ML) tree was constructed using RAxML [41]. The phylogroups of strains were determined by EzClermont [42]. The alignment coverage of SpLE1 in each genome was calculated by blastn with a threshold of >95 % sequence identity using the SpLE1 sequence O157:H7 strain Sakai (no. BA000007) as a reference.

Phylogenetic analyses focused on CC119 were performed using 2 strain sets: 1 including 10 strains each representing ST119 and the 5 SLVs and 4 DLVs of ST119 (indicated in Table S1) and the other including all CC119 strains except for the ST8699 and ST12464 strains (*n*=198). O165:H25 strain JNE072951 (ST119) was used as a reference. For these analyses, SNP sites on PP/IE/IS-free and recombination-free chromosome backbone sequences conserved in all analysed genomes were identified using MUMmer [43] and Gubbins [44] and used for the construction of ML trees with RAxML as previously described [9]. In the construction of a phylogenetic tree of the entire CC119, strains were deduplicated if the recombination-free core sequences were identical. Clustering analysis was performed using fastbaps with ‘optimized BAPs’ in the clustering step [45], and clades were defined at the first level of hierarchical clustering. The ML trees are displayed using iTOL [46] or FigTree v1.4.4 (http://tree.bio.ed.ac.uk/software/figtree/).

### Analyses of the *stx* and *eae* genes, *ter* gene clusters, plasmid replicons and repertoires of T3SS effectors and plasmid-encoded virulence genes

The *stx* subtypes were determined by blastn as previously described [47]. In draft genomes, when both *stx2a* and *stx2c* genes are present, fragmented *stx2* genes are detected. Therefore, we first determined the subtypes of *stx* in assembled sequences by blastn using the reference sequences of each subtype [1] with a threshold of >98 % identity and >99 % coverage. When the top hit showed >50 % coverage to a subtype, the presence of the *stx* gene of this subtype was determined by short-read mapping using SRST2, and the top hit sequence was identified by blastn analysis as a reference with a threshold of >99 % identity and 100 % coverage. As raw read sequence data were not available for five strains, their *stx* subtypes were determined based on the results of the blastn analysis with a threshold of >98 % identity and >50 % coverage. The *eae* subtypes were determined by blastn search (>99 % identity and >90 % coverage) using a previously described reference sequence set [48]. The presence of *ter* genes (*terW*/*Z*/*A*/*B*/*C*/*D*/*E*/*F*) was also determined by blastn (>90 % identity and >99 % coverage) using the sequence of the O157:H7 strain Sakai (ECs1351-6 and ECs1358 in BA000007) as a reference. Plasmid replicons were identified using the MOB-typer algorithm in MOB-suite (v3.1.0) [49].

T3SS effector repertoires in the 13 closed genomes were analysed by blastx as previously described [50]. To detect genes for T3SS effectors and plasmid-encoded virulence factors in draft genomes, intact effector genes and plasmid-encoded virulence genes identified in the 13 closed genomes were clustered using CD-HIT [51] with a threshold of >90 % identity and >30 % coverage. Then, an in-house database including the representative sequences of each cluster was created (the list is shown in Table S4), and the presence of these genes (gene clusters) was determined by blastn (threshold: >90 % identity and >30 % coverage). In this database, we included two effector genes and several virulence genes encoded by the pO157 plasmid of strain Sakai, as they were absent from the 13 closed genomes (*cif*, B171_GEI0.81_09 in AB426049; *espV*, ECRM13516_2756 in CP006262; *stcE*, T2SS genes, *efa1*, ECs_p01, ECs_p02-14 and ECs_p63 in AP018692). The genes included in this database are listed in Table S4.

### Analysis of Stx phages

In the 37 CC119 strains that were available in our laboratory, the presence of Stx2a phages at the *sapB* locus, Stx2c phages at the *prfC* locus and Stx1a phages at the *prfC* or *argW* locus was analysed by long PCR followed by Illumina sequencing and assembly of PCR products as described above. Because the Stx1a phage of strain 2409 was not present at these loci, this integration site was determined by analysing three known integration sites (*wrbA*, *yehV* and *ssrA*) of Stx1a phages [52] with the same long PCR-based strategy. As illustrated in Fig. S1, the segments of Stx prophage regions at each site were amplified using up to four pairs of primers and subjected to Illumina sequencing to obtain the entire sequence of each Stx phage (see Fig. S1 for the primers used). As the full set of segments was not obtained for the Stx2a phage of strain JNE130574 and the Stx1a phage of strain JNE160313, these phages were not sequenced. Gene annotation of Stx phage genomes was carried out with DFAST when necessary. An ML tree of the 37 CC119 strains whose Stx phages were analysed was constructed using their genome sequences along with the 13 closed genomes described above.

### Determination of Stx2 production levels

Cell lysates were prepared from mitomycin C (MMC; Kyowa Kirin or Wako)-treated bacterial cell cultures as described previously [53]. The Stx2 concentration in each lysate was determined by the fluorescence resonance energy transfer (FRET) system that we recently described [53], except that Verotoxin-2 (nacalai tesque) was used as the Stx2 standard.

### Analyses of biochemical characteristics and the *rha* and *csc* gene clusters

The carbohydrate-fermenting abilities of 37 O165:H25 strains (indicated in Table S1) were examined as previously described [18], except that the cultures were monitored for 6 days instead of 2 days, which is standard in this type of examination. Examination of rhamnose and sucrose fermentation on Difco MacConkey agar base (Becton Dickinson) was performed by spotting 3 µl of bacterial suspensions (3.3×10^8^ c.f.u. ml^−1^) onto Difco MacConkey agar base supplemented with 1.0 % (wt/vol) l-rhamnose (nacalai tesque) or sucrose (Wako) and incubating them for 48 h at 37 °C. *E. coli* K-12 strain MG1655 and O26:H11 strain 11 368 were used as positive and negative controls, respectively. Red microcolonies on MacConkey agar base supplemented with sucrose were subcultured on the same agar plate for 16 h at 37 °C. Total cellular DNA was extracted from red and translucent colonies (sucrose-fermenting and nonfermenting colonies, respectively) by the alkaline boiling method and amplified by PCR with the following primers: 5′-CGCAACGCCATAATCACAACAC-3′ and 5′-GATGTCGTCAAGCTCTCGGAA-3′. The sequence of the amplicon was determined by the Sanger method using the same primers.

To determine the presence/absence and sequences of seven l-rhamnose metabolism-associated genes (*rhaM*/*D*/*A*/*B*/*S*/*R*/*T*), four sucrose metabolism-associated genes (*cscB*/*K*/*A*/*R*) and one fructokinase-encoding gene (ECO26_4356) in CC119 strains, we searched for these genes in 202 CC119 genomes by blastx (>95 % identity and 100 % coverage) using the sequences of strain MG1655 (b3901-b3907 in NC_000913), strain EC3132 (X81461) and strain 11 368 (AP010953) as references. When a gene showed low coverage (<100 %), we examined the presence of insertions or deletions (InDels) in the gene and determined the positions of InDels by blastn if present.

### Statistical analysis

Using 82 clinical isolates sequenced in this study, Fisher’s exact test [54] with Benjamini–Hochberg correction [55] was performed for comparison of incident rates of severe disease (defined as bloody diarrhoea or HUS) between the entire dataset and each of the 4 clades that included >10 strains.

## Results and discussion

### Strain set

We sequenced 80 O165:H25 strains (77 Japanese and 3 Belgian strains) and 10 O172:H25 strains (all Japanese strains) in this study. The major ST of O165:H25 in our strain set was ST119, and all O172:H25 strains belonged to an SLV of ST119 (ST660). We therefore collected publicly available genome sequences belonging to the SLVs (ST660/5486/7316/8250/13714) and DLVs (ST8699/12464/13715/13560) of ST119. After excluding low-quality sequences, the final set included the genome sequences of 202 strains from various geographical regions (see Table S1 for details). In this study, we defined these strains as CC119.

The majority of strains (*n*=161) belonged to ST119, with ST660 being the second major ST (*n*=20) (Table 1). The major serotype was O165:H25 (*n*=166), followed by O172:H25 (*n*=19) and ONT:H25 (*n*=15). The remaining two were O76:H25 (both belonged to ST12464). Although Japanese and US strains represented 43 and 41 % of the strain set, respectively, strains isolated in other countries, such as Canada (*n*=6) and European countries (*n*=18), were also included. Most strains (*n*=185) were human isolates, while others (*n*=17) were isolated from various sources: animals (bovine and wild animals), foods (cilantro, cheese and the like) and the environment. The strain set included strains showing various *stx* genotypes, while two ST12464 strains were *stx*-negative.

**Table 1.** CC119 strain set analysed in this study ST2122 (double-locus variant of ST119), for which no genome sequences were available, was not included.

		Sequence type (ST)										
		ST119	ST660*	ST5486*	ST7316*	ST8250*	ST13714*	ST13715†	ST13560†	ST8699†	ST12464†	Total
Serotype												
	O165:H25	154	0	4	4	1	1	1	0	1	0	166
	O172:H25	0	17	0	0	0	0	0	2	0	0	19
	O76:H25	0	0	0	0	0	0	0	0	0	2	2
	OUT:H25	7	3	0	4	0	0	0	0	1	0	15
Country												
	Japan	71	8	1	3	0	1	1	2	0	0	87
	USA	72	8	1	1	1	0	0	0	0	0	83
	Canada	4	0	2	0	0	0	0	0	0	0	6
	Eur. countries	12	4	0	0	0	0	0	0	0	2	18
	Australia	1	0	0	4	0	0	0	0	2	0	7
	South Africa	1	0	0	0	0	0	0	0	0	0	1
Source												
	Human	148	19	3	8	1	1	1	2	1	1	185
	Animal	4	0	1	0	0	0	0	0	0	1	6
	Food	5	1	0	0	0	0	0	0	0	0	6
	Environment	4	0	0	0	0	0	0	0	0	0	4
	No information	0	0	0	0	0	0	0	0	1	0	1
*stx* genotype												
	*stx2a*	25	18‡	0	0	0	0	0	2	0	0	45
	*stx2c*	0	0	0	0	0	0	0	0	2	0	2
	*stx1a*/*stx2a*	73	2	4	0	0	0	1	0	0	0	80
	*stx1a*/*stx2c*	0	0	0	1	0	0	0	0	0	0	1
	*stx2a*/*stx2c*	61	0	0	1	1	1	0	0	0	0	64
	*stx1a/stx2a*/*stx2c*	2	0	0	6	0	0	0	0	0	0	8
	Negative	0	0	0	0	0	0	0	0	0	2	2

*Single-locus variants of ST119.

†Double-locus variants of ST119.

‡One strain (90-3040) contained two *stx2a* genes.

European countries (*n*=18): United Kingdom (*n*=11), Belgium (*n*=3), Germany (*n*=2), France (*n*=1), Norway (*n*=1).

UT, untypeable.

### Position of CC119 in the *E. coli* phylogeny and phylogenetic relationships between the strains belonging to different STs in CC119

**Fig. 1.**
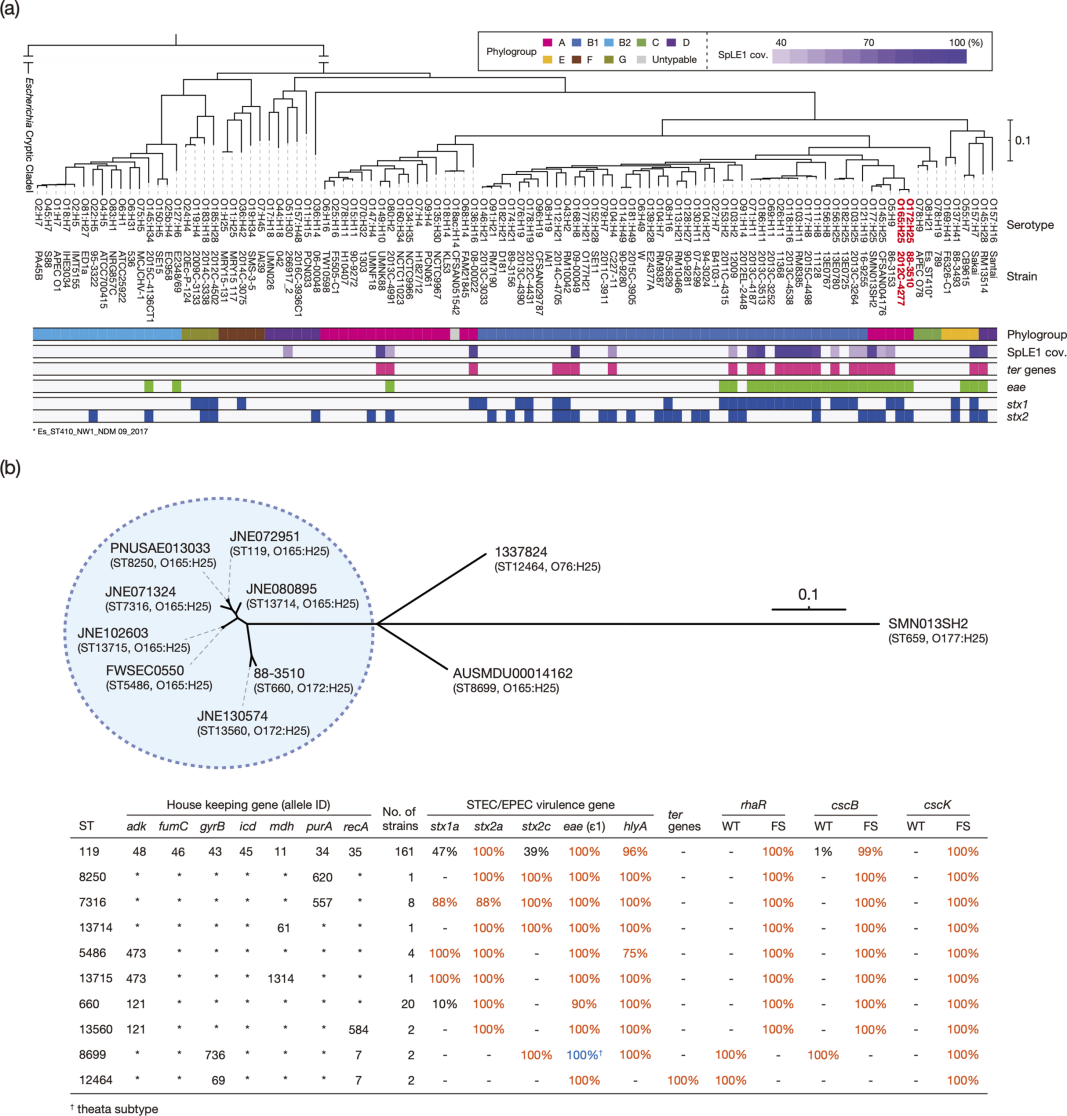
Phylogenetic position of CC119 in *E. coli* and STEC-related gene profiles of CC119 strains. (**a**) A core gene-based maximum-likelihood (ML) tree of 104 chromosome-closed *E. coli* strains with an *Escherichia* cryptic clade I strain (TW10509; AEKA00000000) as an outgroup is shown along with the names and serotypes of each strain. The tree was constructed based on the 100 652 SNPs identified in 2647 core genes. The phylogroups, the alignment coverages of the SpLE1 sequence of O157:H7 strain Sakai [10] and the presence (coloured) or absence (open) of the *ter* gene cluster and the *eae*, *stx1* and *stx2* genes in each strain are also indicated. The percentage coverage (>99 % nucleotide sequence identity threshold) of the SpLE1 sequence is indicated by the colour gradient. The *ter* gene cluster, which comprises eight genes, was judged as ‘positive’ when seven or eight genes were detected. (**b**) An unrooted ML tree of CC119 strains, each representing 10 STs, is shown with an O177:H25 strain as an outlier. The tree was constructed based on the recombination-free SNPs (*n*=9,263) identified on the chromosomal backbone (3 864 656 bp). The strain names are displayed at each tip with their STs and serotypes. ST119 and its close relatives (defined as CC119ss in the main text) are indicated by a blue circle. In the lower panel, allele IDs for MLST and the prevalence of the *stx1*, *stx2*, *eae*, *hlyA* and *ter* genes in each ST are shown, along with that of the wild type (WT) or frameshift (FS) mutation-containing *rhaR*, *cscB* and *cscK* genes. A high prevalence (>80 %) is indicated by coloured characters. The presence/absence of *ter* genes was determined as described in (**a**). Bar, the mean number of nucleotide substitutions per site.

To examine the phylogenetic position of CC119 in *E. coli *, we selected two strains representing each of the two major STs of CC119 (ST119 and ST660) and constructed a core gene-based phylogenetic tree along with a strain set covering the entire *E. coli* as much as possible (listed in Table S3). The ST119 (O165:H25) and ST660 (O172:H25) strains formed a cluster with three STEC strains of serotypes O5:H9, O145:H25 and O177:H25 (Fig. 1a), which emerged from the common ancestor with O121:H19, one of the major STEC serotypes [9].

To analyse the phylogenetic relationship between the STs in CC119, we selected strains representing each ST (one strain for one ST) and constructed a WGS-based phylogenetic tree (Fig. 1b). All SLVs clustered with ST119, as indicated by the blue circle in Fig. 1b. ST13715 (SLV of ST5486) and ST13560 (SLV of ST660) were also included in this cluster. However, two DLVs (ST8699 and ST12464) each formed distinct branches, indicating that they were distantly related to the strains in the cluster, although the serotype of ST8699 was O165:H25. Therefore, we focused subsequent analyses principally on the strains (*n*=198) belonging to this cluster named CC119 *sensu stricto* (CC119ss). The serotype of ST8699 (O165:H25) suggests that the ancestral serotype of CC119ss was O165:H25.

### Distribution of STEC major virulence-related genes in CC119

Examination of the distribution of STEC major virulence-related genes (*stx1*, *stx2*, *eae* and *hlyA*) revealed that the *stx2a*, *eae* (subtype ε1) and *hlyA* genes were present in almost all CC119ss strains, while *stx1a* and *stx2c* were variably present in CC119ss (the summary table in Fig. 1b). This finding suggests that the Stx2a phage, the LEE and the virulence plasmid were acquired by the common ancestor of CC119ss. Although both ST8699 strains contained the *stx2c*, *eae* and *hlyA* genes, they lacked *stx2a,* and the subtype of their *eae* genes was θ. As both ST12464 strains only contained the *eae* gene (ε1), they were determined to be enteropathogenic *E. coli* (EPEC).

### Phylogenetic and evolutionary overview of CC119ss

**Fig. 2.**
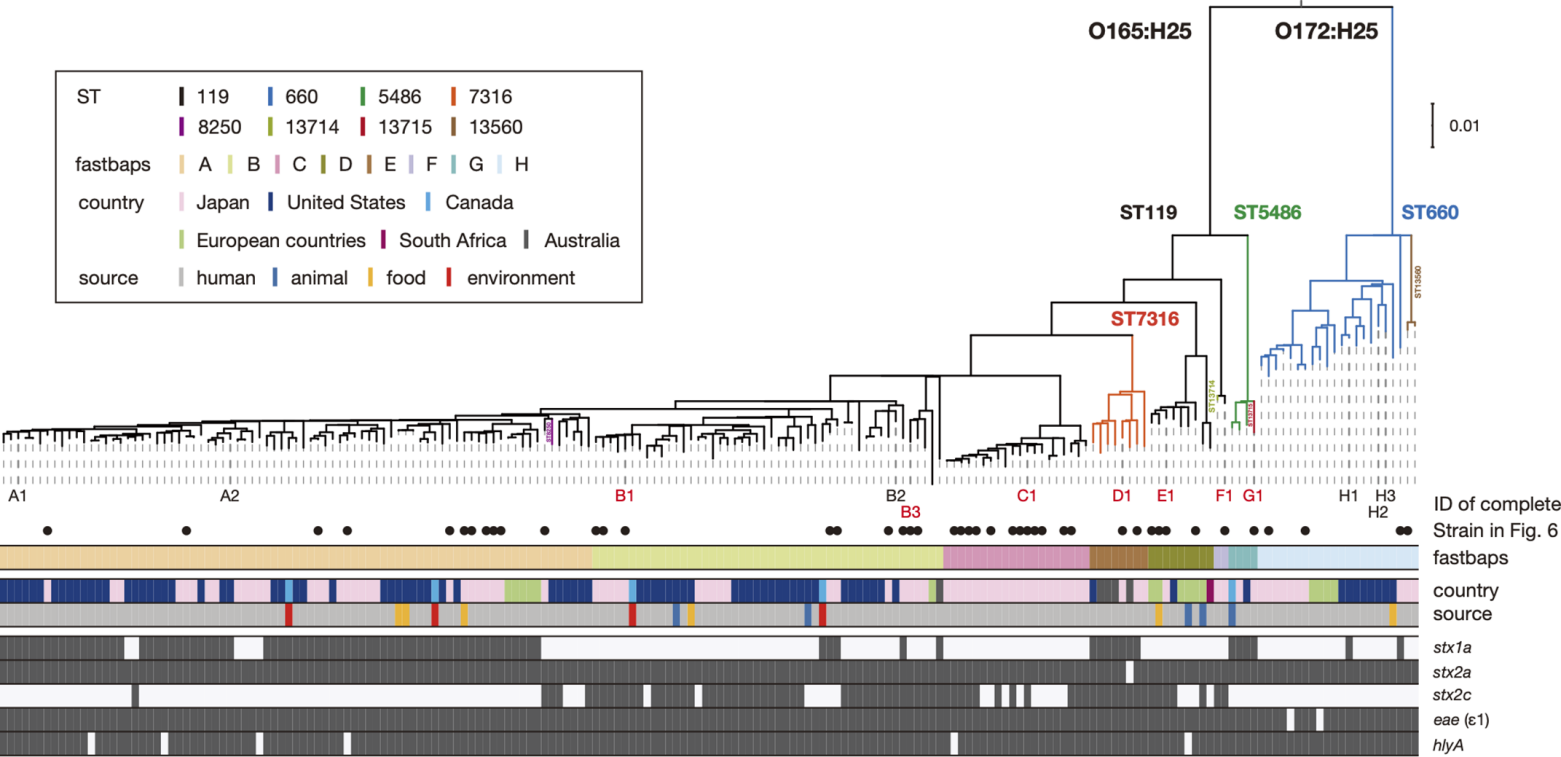
Phylogenetic relationships of 194 CC119ss strains. A maximum-likelihood (ML) tree constructed based on the recombination-free SNPs (*n*=8,471) identified on the chromosomal backbone (3 669 646 bp) is shown along with strain information on fastbaps clades; isolation countries and sources; and the presence/absence of *stx1a*, *stx2a*, *stx2c*, *eae* (subtype ε1) and *hlyA* genes. The positions of 13 genome-closed strains are indicated by IDs (A1–H3). Of the 13 strains, the 7 strains sequenced in this study are indicated in red. The presence and absence of the five genes are indicated by filled and open boxes, respectively. Bar, the mean number of nucleotide substitutions per site.

WGS-based phylogenetic analysis of 198 CC119ss strains revealed that CC119ss comprises eight distinct clades (A–H) defined by fastbaps-based clustering (Fig. 2). This analysis also suggests that clade H (ST660 and ST13560) was the first separated in the CC119ss lineage, followed by clades G (ST5486 and ST13715), F (ST119 and ST13714), E (ST119), D (ST7316) and C (ST119), in that order; finally, clade A (ST119; including a sole ST8250 strain) emerged from clade B (ST119). Although most clades (A, B, D, E, G and H) contain strains from various geographical regions, clade C only comprises Japanese strains and thus can be regarded as a Japan-specific sublineage. Although the strain set included small numbers of strains from nonhuman sources, these strains were distributed among human strains in five clades. Biochemical characteristics of the CC119ss strains and some other factors might affect the variability of our strain set because a previous study using bovine O165:H25 isolates in German cattle farms suggested the presence of a bovine-specific subpopulation [56], as in the STEC O157:H7 lineage [57, 58]. Therefore, we cannot exclude the possibility of exclusion or underrepresentation of lineages that are less likely to cause overt disease in humans from our strain set. To understand a more complete population structure of the CC119ss, more variable isolates, especially bovine isolates, need to be analysed. In addition, as the geographical origins of the isolates were biased towards Japan and the USA, more strains isolated in other geographical regions also need to be analysed.

Taking advantage of the clinical information of 82 Japanese or Belgium strains sequenced in this study, we analysed the difference in the incident rates of severe disease (defined as bloody diarrhoea or HUS) between clades (only clades A, B, C and H that included >10 strains were analysed), but none of the 4 clades showed a significantly higher rate than the average of the entire strain set (*P*>0.05 by Fisher’s exact test after false discovery rate correction by the Benjamini–Hochberg method).

### General features of the closed genomes

While closed genome sequences were publicly available for six strains [59, 60], they belonged to three of the eight clades (A, B and H) (Fig. 2). To gain a more complete genomic view of the entire CC119ss lineage, particularly that of MGEs in CC119ss, we determined the complete genome sequences of seven strains, five representing each of the other five clades and two belonging to clade B, which is relatively understudied. This allowed us to perform detailed analyses of a total of 13 closed genomes covering the entire CC119ss lineage (these strains were referred to as simple IDs linked with their clades, from A1 to H3). As summarized in Table 2, the chromosomes of the 13 strains range from 5207 to 5429 kb in size and contain various numbers of PPs (18–22), IEs (3 each) and plasmids (1–4). Seven strains contain transposable Mu-like phages (1–3 per genome), but they were analysed separately from the PPs in this study.

**Table 2.** List of the 13 closed genomes analysed in this study

					Genome size (kb)			no. of						
Strain ID in this study	Strain name	Serotype	ST	fastbaps clade	Total	Chromosome	Plasmid	PPs	IEs	Mu-like phages	IS elements	Plasmids	Accession no.	Reference
A1	2013 C-4830*	O165:H25	ST119	A	5303	5136	93/75‡	19	3	0	125	2	CP027325-7	[60]
A2	2012 C-4277†	O165:H25	ST119	A	5375	5203	98/75‡	21	3	0	119	2	CP013028-30	[59]
B1	JNE072951	O165:H25	ST119	B	5268	5159	77‡/33	19	3	0	124	2	AP026739-41	This study
B2	88–3001*	O165:H25	ST119	B	5270	5196	75‡	19	3	1	121	1	CP027363-4	[60]
B3	JNE110611	O165:H25	ST119	B	5314	5174	75‡/61/4	19	3	1	123	3	AP026742-5	This study
C1	PV06-71	O165:H25	ST119	C	5283	5077	112/80‡/7/7	18	3	1	127	4	AP026746-50	This study
D1	JNE071324	O165:H25	ST7316	D	5429	5285	76‡/68	22	3	1	131	2	AP026751-3	This study
E1	PV01-97	O165:H25	ST119	E	5420	5165	98/77‡/41/40	21	3	0	119	4	AP026754-8	This study
F1	JNE072929	O165:H25	ST119	F	5306	5230	75‡	19	3	3	121	1	AP026759-60	This study
G1	JNE102603	O165:H25	ST13715	G	5383	5307	76‡	20	3	2	126	1	AP026761-2	This study
H1	2013 C-3492*	O172:H25	ST660	H	5270	5196	74‡	20	3	0	126	1	CP027445-6	[60]
H2	90–3040*	O172:H25	ST660	H	5328	5254	74‡	22	3	1	128	1	CP027459-60	[60]
H3	88–3510*	O172:H25	ST660	H	5207	5140	66‡	20	3	0	130	1	CP027675-6	[60]

*Annotated using DFAST in this study.

†Reannotated using DFAST in this study

‡STEC virulence plasmid

IE, integrative element; IS, insertion sequence; PP, prophage; ST, sequence type.

**Fig. 3.**
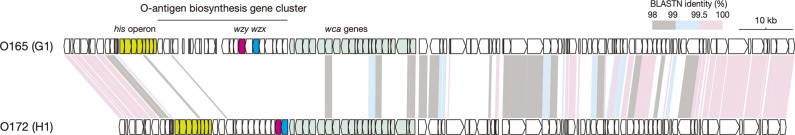
Comparison of the O-antigen biosynthesis gene clusters and their flanking regions between O165:H25 and O172:H25. Genetic organizations of the O-antigen biosynthesis gene cluster and its flanking regions in strains G1 and H1 representing each O-serotype are shown. The levels of nucleotide sequence identities between CDSs are indicated by coloured shading.

The chromosome backbone was well conserved among the 13 strains, while large inversions were found in several strains (Fig. S2). All these inversions were classified into two types: (i) inversion across the termination of replication (*ter*) via recombination between similar prophage regions and (ii) IS*629*-mediated inversion across the origin of replication (*ori*). As expected, the O-antigen biosynthesis gene clusters (OAGCs) showed a clear difference between the O165 and O172 strains (Fig. 3). The regions showing notable sequence variation extended to the *his* operon at one side and to the region approximately 52 kb downstream of the colonic acid biosynthesis gene cluster (*wca* genes) at the other side, suggesting that large genomic recombination induced the O-antigen switch from O165 to O172 [61–63]. Mosaic patterns observed in the region downstream of *wca* genes suggest a complex evolutionary history of this region before or after the recombination event.

### Variation in MGEs in the closed CC119ss genomes

#### Integration sites of PPs/IEs

**Fig. 4.**
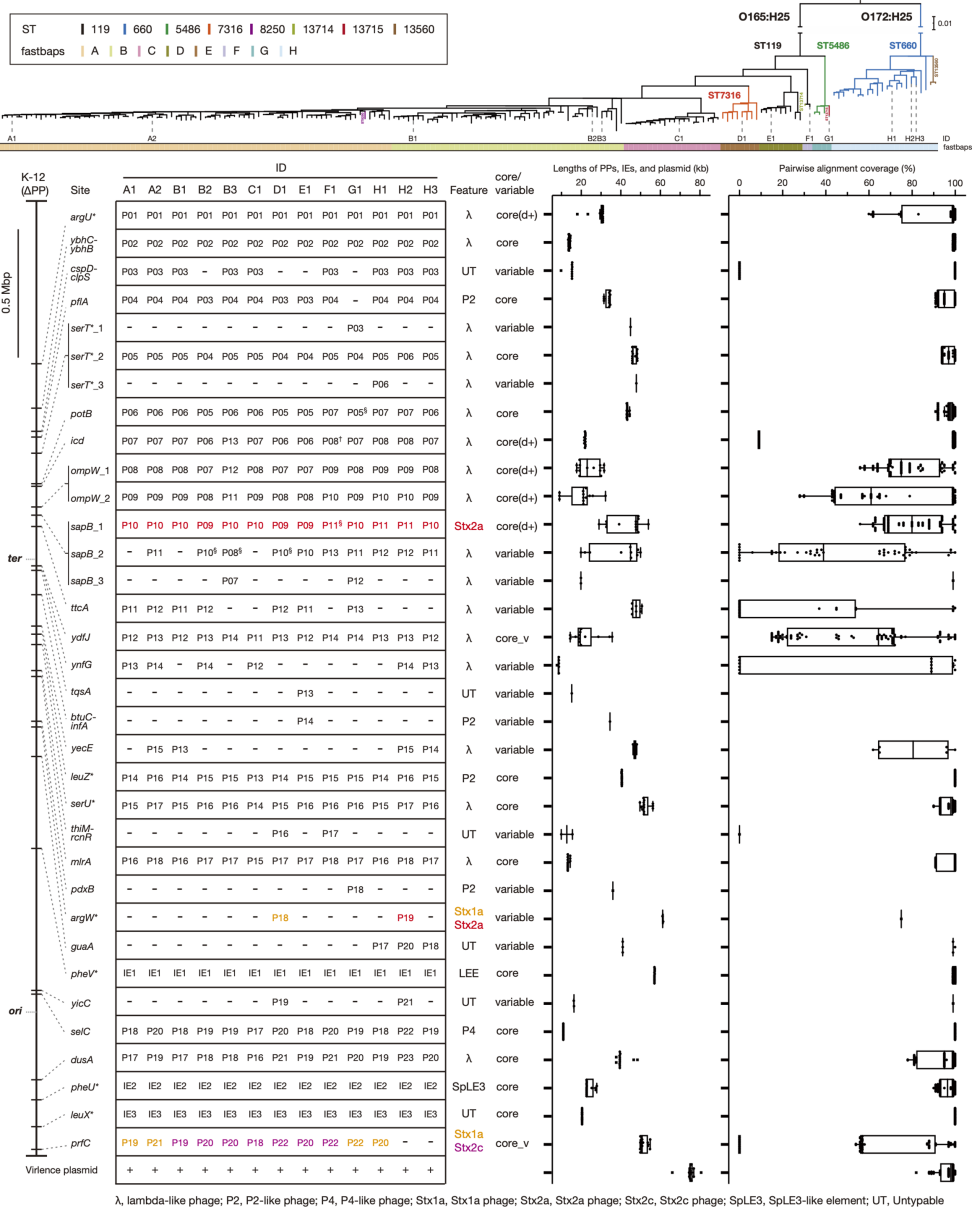
Conservation and variation of the prophages (PPs), integrative elements (IEs) and virulence plasmids in the 13 closed CC119ss genomes. In the upper panel, the positions of the 13 genome-closed strains are indicated in the maximum-likelihood (ML) tree (the same tree as in Fig. 2). In the lower panel, the chromosomal integration sites of PPs and IEs identified in the 13 genomes are shown on the PP-removed chromosome backbone of strain K-12 MG1655 (K-12∆PP; asterisks indicate tRNA genes) on the left-hand side, while the presence/absence of PP/IE at each site in each strain, the features of each PP, the types of integration sites, the length distributions and pairwise alignment coverage (with >99 % nucleotide sequence identity) of PPs/IEs integrated into each site are shown on the right-hand side. The latter two data are shown by box-and-whisker plots with the median (line in box), first and third qualities (edges of box), and 1.5× interquartile range (lines extending from edges of box). Outliers are indicated by black squares. The data for the virulence plasmid are also shown at the bottom. The highly degraded PP at *icd* of strain F1 (marked by a dagger; see Fig. S3 for its genome structure) is not indicated in the plot. Mu-like phages integrated into several PPs (indicated by section) were excluded from the analyses of PP lengths and pairwise alignment coverage. In the column ‘core/variable’, those containing PPs/IEs in >75 % strains are indicated by ‘core site’, and the remaining are indicated by ‘variable site’. The core sites are divided into three groups (core, core(d+) and core_v) according to the variations in lengths and genome sequences (see the main text for more details).

We identified a total of 34 integration sites for PPs and IEs (31 for PPs and 3 for IEs) in the 13 closed genomes (Fig. 4 and Table S5: note that the 13 bp *attB* sequence for the PP at *sapB* overlaps the coding sequence of *sapC* and that the integration sites of Mu-like phages are excluded here). At three loci, two or three PPs were inserted in tandem. In this study, integration sites at these loci were distinguished based on the integration pattern of PPs (*serT*_1–3) or the order of PP integration (*ompW*_1 and 2 and *sapB*_1–3). Among these integration sites, 18 were occupied by PPs/IEs in most strains (11–13 strains); thus, they were defined as ‘core sites’, and the remaining 16 sites were defined as ‘variable sites’ in this study.

#### PPs

All-to-all comparison of the identified PPs (excluding Mu-like phages) revealed that PP genomes integrated into most core sites were well conserved as judged by the high proportion of shared genome sequences (>99 % identity) (Fig. 4), while deletions of various lengths were detected in five sites [*argU*, *icd*, *ompW*_1, *ompW*_2 and *sapB*_1; indicated by core(d+) in Fig. 4] (see Fig. S3 for details). The exceptions were the PPs at *ydfJ* and *prfC* (indicated by core_v in Fig. 4), which showed notable variation in genome sequence. While the variation in the PPs at *ydfJ* appeared to be caused by intrachromosomal recombination with PPs having similar sequences (Fig. S2), that of the PPs at *prfC* was introduced by the exchange of Stx1a or Stx2c phages as described in a later section. The presence of PPs at 16 variable sites (mostly in 1 or a few strains) indicated frequent acquisition and loss of PPs in CC119. Taken together, these findings revealed that while many PPs were acquired by the common ancestor of CC119ss and have been stably maintained in most cases, notable alterations in PP content also occurred in the CC119ss lineage.

#### IEs

All IEs at core sites were highly conserved in size and sequence (Fig. 3). The LEE was present at *pheV,* and its core region encoding a set of T3SS genes was similar to that of the major STEC serotype strains [4, 8, 9], but the integrase gene was missing, and the *espP* gene was present in the accessory region unique to CC119ss (Fig. S4). The high conservation of LEE sequences is consistent with the notion that the LEE was acquired by the common ancestor of CC119ss.

#### Plasmids

Virulence plasmids were also highly conserved in size and sequence except for the plasmids of strains C1 and H3, which contained an insertion or deletion of a 5 or 9 kb sequence, respectively (Table 2 and at the bottom of Fig. 4). In addition to the *ehx* and *ecf* operons and the *katG* and *espP* genes that are present in the virulence plasmids of STEC O157:H7 and other major non-O157 STEC [8, 9, 64, 65], the virulence plasmids of CC119ss encode the *sfp* operon for the biosynthesis of Sfp fimbria identified in the plasmid of the sorbitol-fermenting STEC O157:H- strain as previously reported [28, 66] (Fig. S5a).

Additional plasmids were found in 7 of the 13 strains (up to 3 plasmids). These plasmids were variable in size (3.8–112 kb) and sequence (Figs S5b and S5c), even between plasmids with the same type of replicon (Fig. S5c). Although these plasmids did not appear to be involved in the virulence of each strain, as no virulence-related genes were found (Fig. S5b), streptomycin resistance genes (*strAB*) were found in the 112 kb plasmid in strain C1.

A plasmid replicon search of the entire CC119ss strain set revealed that although the 2 replicons of the virulence plasmid (*IncFIB* and *IncFIA*) were highly conserved, the other 29 replicons identified by this search were sporadically distributed (Fig. S6). This result indicates the high conservation of virulence plasmids and the frequent gain and loss of other plasmids in CC119ss, similar to the observation in the O145:H28 and O121:H19 STEC lineages [8, 9]. Notably, the replicon (rep_cluster_2350) of a small plasmid carried by strain C1 (pO165_PV06-71_3) was found in 95 % of the strains belonging to clade C, the Japan-specific clade, indicating the stable maintenance of pO165_PV06-71_3 in this clade, which is probably related to the geographical distribution of clade C specific to Japan (Fig. 2).

### Mu-like phages and IS elements

Among the 10 Mu-like phages identified in the 13 closed genomes, 6 were inserted into MGEs, and the others were found in various chromosomal loci (Fig. S7a). The genome sequences of these phages were well conserved and exhibited overall sequence similarity to Sp18 of the O157:H7 strain Sakai (Fig. S7b). The variable location of Mu-like phages observed is consistent with the transposition-dependent replication of the Mu-like phage family [67].

The total copy numbers and repertoires of IS elements were also similar among the 13 closed genomes (Table 2 and Table S6), with IS*2*, IS*600* and IS*629* being the major IS species (28–32, 23–33 and 31–40 copies/genomes, respectively). Some interstrain variation was found for the copy number of each IS, particularly for those of the three major ISs, but clear within-clade variation was not observed. This may suggest a relatively reduced activity of IS transposition in the CC119ss lineage.

### Repertoires of T3SS effectors and plasmid-encoded virulence factors in CC119ss

In the 13 closed genomes, we identified many genes encoding T3SS effectors belonging to 26 families (42–53 copies per genome; see Table S7 for details). The effector repertoires of CC119ss strains were very similar to each other and to those of major STEC [4, 8, 9]. Almost all (25/26) T3SS effector families were found on the PPs/IEs integrated into core sites (Table S5). The only exception was the *espW* gene encoded by the PPs integrated into *yecE*, one of the variable sites, but homologues of other effectors encoded by the PPs at *yecE* (EspM, NleC, NleG, OspB and TccP2) were encoded by other PPs/IEs integrated into core sites.

**Fig. 5.**
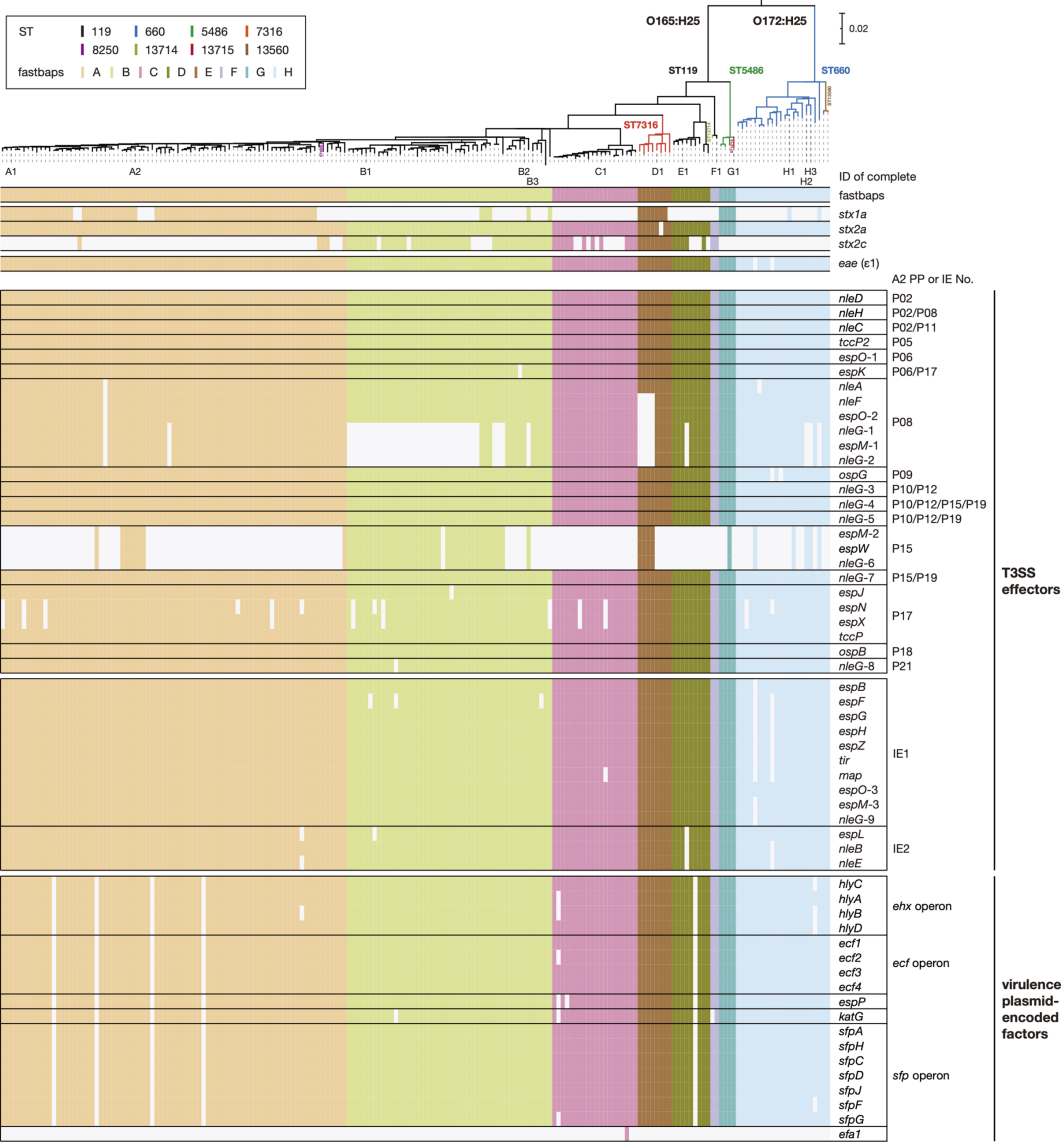
Distribution of major virulence-related genes in CC119ss strains. Along with the maximum-likelihood (ML) tree of the 194 CC119ss strains (the same tree in Fig. 2), the presence or absence of each subtype of *stx* (*stx1a*, *stx2a*, or *stx2c*), PP- and IE-encoded T3SS effector genes, and virulence plasmid-encoded virulence genes in each strain are shown. The PPs and IEs of strain A2 are indicated, as this strain contained the highest number of T3SS effector genes among the 13 genome-closed strains. Bar, the mean number of nucleotide substitutions per site.

Analysis of the entire CC119ss lineage also revealed the high conservation of the T3SS effector genes identified in the closed genomes except for two sets of effector genes (*nleG*-1/*espM*-1/*nleG*-2 and *espM*-2/*espW*/*nleG*-6) (Fig. 5). The former set was absent from many strains in clade B and several strains in other clades. This distribution pattern was probably generated by the deletion of a segment in the PP genome at *ompW*_1, as observed in strain H3 (Fig. S3). The latter set, which is encoded by the PPs at *yecE*, was present in many strains in clade B but sporadically distributed in other clades, consistent with the variable distribution of PPs at *yecE*. Plasmid-encoded virulence genes found in closed genomes were also highly conserved in the entire CC119ss population (Fig. 5). These results indicate that many of the T3SS effector and plasmid-encoded virulence genes were acquired by the common ancestor and have been stably maintained in CC119ss, and this finding is apparently linked to the acquisition process and stable maintenance of MGEs (PPs/IEs/plasmid) encoding these genes in the CC119ss lineage.

### Variation in the Stx phages in CC119ss

**Fig. 6.**
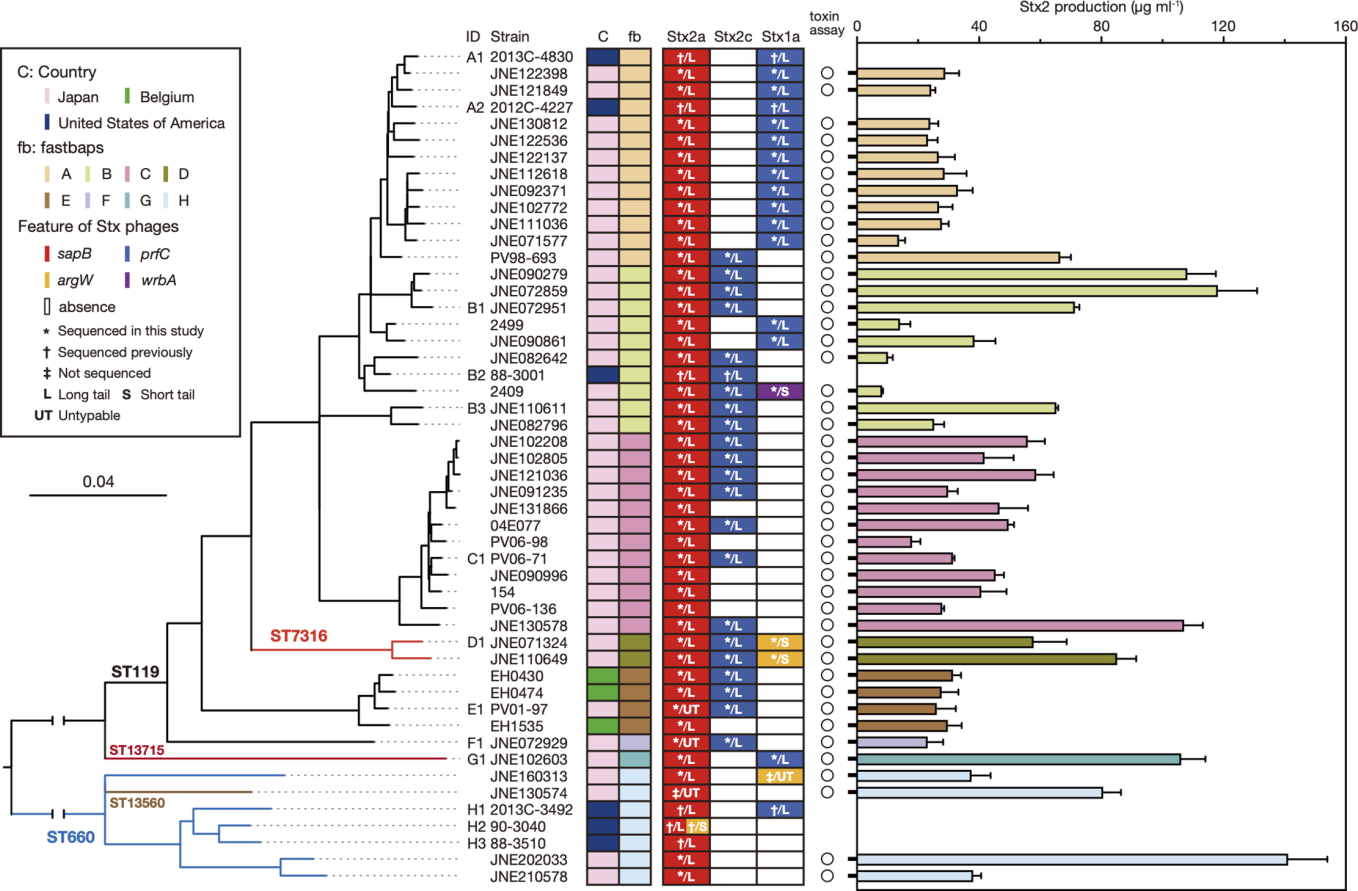
Variation in the integration sites of Stx phages and the Stx2 production levels among CC119ss strains The maximum-likelihood (ML) phylogenetic tree of 50 CC119ss strains, which was constructed based on the recombination-free SNPs (5165 sites) identified on the conserved chromosome backbone (4 129 840 bp), is shown, along with the geographic and clade information concerning strains and the presence/absence, integration sites (coloured box in each prophage) and types (long- or short-tailed) of Stx prophages. The IDs of 13 genome-closed strains are also indicated. Strain 90–3040 (**H2**) contained two Stx2a phages at *sapB* and *argW*. In the right panel, the MMC-induced Stx2 production levels of each strain are shown as the mean values with standard deviations of biological triplicates. Note that the Stx2 production levels of six *stx2*-positive strains whose closed genome sequences were obtained from NCBI were not determined.

The CC119ss strains analysed in this study contained *stx1a, stx2a* and *stx2c* in combination. Among these, *stx2a* was highly conserved in the CC119ss lineage (Figs 1b, 2 and 5). In contrast, *stx1a* and *stx2c* were variably distributed, showing somewhat clade-dependent manners (*stx1a*-positive strains were enriched in A, D and G, while *stx2c*-positive strains were enriched in B, C, D, E and F), with only eight strains carrying both *stx1a* and *stx2c* (two in clade B and six in clade D). To better understand the variation and evolutionary history of Stx phages in CC119ss, we selected 37 strains available in our laboratory (Table S2) so that they, together with the 13 genome-closed strains, covered the entire CC119ss lineage as much as possible (Fig. 6) and determined the integration sites and genome sequences of their Stx phages by a long-PCR-based strategy, as illustrated in Fig. S1. Although two Stx phage genomes (the Stx2a phage of strain JNE130574 and the Stx1a phage of strain JNE160313) were not determined, we obtained full-length sequences of the remaining Stx phages, allowing detailed analyses of a total of 91 Stx phage genomes (50 Stx2a, 22 Stx2c and 19 Stx1a phages; Fig. 6). Insertion of Mu-like phages was identified in several Stx phage genomes (Table S2), but the sequences of Mu-like phages were excluded from the subsequent sequence comparison of Stx phages.

All strains contained Stx2a phages at *sapB,* and they exhibited a high level of sequence similarity (Fig. S8), although IS mediated-short inversion (four clade C strains) and deletion of the entire or parts of late genes (one clade E strain, the clade F strain and all clade H strains) were observed. This finding supports our hypothesis that the integration of the Stx2a phage was an ancestral event. Although strain H2 contained an additional Stx2a phage at *argW*, this phage was clearly different from the Stx2 phages at *sapB*; those at *sapB* were long-tailed phages with late genes similar to those of phage lambda, whereas the Stx2 phage at *argW* was a short-tailed phage similar to the Stx2a phages of O157:H7, such as Sp5 [68] and 933W [69].

Stx1a and Stx2c phages exhibited variable distribution, but all Stx2c phages were integrated into the *prfC* locus (Fig. 6), and their genome sequences were highly conserved (Fig. S9a), suggesting that the Stx2c phage was already present, at least in the common ancestor of clades A–F (Fig. 5). In contrast, Stx1a phages were found at the *prfC*, *argW*, or *wrbA* loci (Fig. 6). While most Stx1a phages were found at *prfC* (*n*=16) and all were long-tailed phages with a highly conserved genome sequence (Figs S9a, S9b), Stx1a phages at *argW* and *wrbA* were only present in two and one strains, respectively. In addition, the Stx1a phages found at *argW* and *wrbA* were short-tailed phages but had distinct genome sequences (Fig. S9c). Considering the phylogenetic positions of *stx1a*-positive strains (Fig. 6), it is most likely that the Stx1a phages at *prfC* were acquired very early in the diversification process of CC119ss, perhaps by the common ancestor of CC119ss, and that the Stx1a phages at *argW* and *wrbA* were acquired later (Fig. S9a).

With respect to the acquisition history of Stx1a and Stx2c at *prfC*, the possible timing of their acquisition mentioned above suggests that the Stx1a phage was first integrated into *prfC* and then replaced by or changed to the Stx2c phages. In this regard, the high sequence similarity at both ends of the prophage regions between the Stx1a or Stx2c phages favours a scenario in which the preexisting Stx1a phage was changed to Stx2c by recombination (Fig. S9b). However, this phage shift was observed in multiple sublineages in multiple clades and thus does not follow the phylogeny of host strains (Fig. 6 and Fig. S9a). Considering the high sequence conservation between Stx2c phages, recombination between the same or very similar Stx2c phages and the preexisting Stx1a phage might have occurred multiple times in the evolutionary history of CC119ss. However, the probability of such genetic events is very low. Thus, the mechanism underlying the phage shift at *prfC* is currently unknown, although if an Stx2c phage that shares very similar integrase and late genes with the preexisting Stx1a phage was once integrated in tandem into *prfC*, the phage shift could occur multiple times by recombination between the two prophages without leaving clearly recognizable recombination junctions. Complete replacement by a very similar phage (its integration after the deletion of the preexisting phage leaving the *att* site intact) is also possible if such a similar phage is circulating in a specific environment.

### Variation in the Stx2 production level among CC119ss strains

The variation in the MMC-induced Stx2 production level among CC119ss strains was analysed using 44 strains in the strain set used for the Stx phage analysis (Fig. 6 and Table S2). Notable variation was observed, ranging from 8.1 to 141 µg ml^−1^ (average: 45.8 µg ml^−1^). Intraclade variation was also observed in clade B (8.1–118 µg ml^−1^), clade C (17.9–107 µg ml^−1^) and clade H (37.4–141 µg ml^−1^). The homologus time-resolved FRET (HTRF) assay used in this study detects both Stx2a and Stx2c [53]. However, as the level of Stx2c production by STEC strains carrying only *stx2c* is generally very low [70, 71], the observed Stx2 production levels are mostly attributable to Stx2a (note that CC119ss strains carrying only *stx2c* were not available).

The expression of *stx2* strongly depends on the late promoter [72, 73], and Stx2 production is tightly coupled with phage induction. Although a correlation of the variation in Stx2a phage genomes with the Stx2 production levels of host strains was shown in STEC O157 [71], the Stx2 phage genomes in CC119ss strains were highly conserved in sequence as described above (Fig. S8). Thus, very small differences in the sequences of Stx2a phage genomes, even at SNP levels, and/or some other genetic differences between host strains may have generated the observed variation in the Stx2 production level among CC119ss strains.

### Search for biochemical markers for CC119ss strains

Resistance to tellurite is widely used for the screening of STEC [14]. However, we have previously reported that O165:H25 strains are tellurite-sensitive [18]. Consistent with this, the *ter* genes were absent from all O165:H25 genomes analysed, except for two ST12464 strains that do not belong to CC119ss. The *ter* genes were also absent from all O172:H25 genomes (Fig. 1b). Thus, the *ter* genes were missing from the entire CC119ss lineage. The *ter* genes are encoded on SpLE1 or SpLE1-like IEs [4, 8, 9]. A search of the *ter* genes and SpLE1-like IEs in the closed *E. coli* genome set shown in Fig. 1a revealed that strains in the sister branches of CC119, including O121:H19 (Fig. 1a and Table S3), contain *ter* genes and SpLE1-like IEs. This result, together with the presence of these genes in ST12464, indicates that the common ancestor of CC119ss lost the SpLE1-like IE; thus, tellurite resistance cannot be used for the selection of CC119ss strains.

To explore biochemical markers useful for the detection and identification of CC119ss strains, we first examined the ability of 37 O165:H25 strains to ferment 14 carbohydrates using Andrade’s peptone water containing each carbohydrate (1 % wt/vol) (Table 3). In this screening, positive signs of rhamnose and sucrose fermentation were only observed after 2 days or longer of incubation and there were no signs of salicin and dulcitol fermentation, in contrast to the positive reactions for these carbohydrates that are usually observed within 2 days in *E. coli* [74]. Thus, these types of fermentation could be used as biochemical markers for CC119. Using 13 closed genomes, we analysed 4 gene sets that are known to be involved in the uptake and degradation of l-rhamnose (*rha* genes) [75, 76], sucrose (*csc* genes) [77, 78], salicin (*bgl* operon) [79] and dulcitol (*gat* operon) [80] in *E. coli* and found that all genomes contained 1 or 2 apparently inactivated genes in each gene set (see Table S8 for the details of mutations). In addition, the *scr* genes encoding a sucrose-specific phosphotransferase system (PTS) [78] were absent from all genomes. These genotypic traits were apparently responsible for the defects in the fermentation of the 4 carbohydrates observed for 37 O165:H25 strains.

**Table 3.** Carbohydrate fermentation abilities of 37 O165:H25 strains

		Cultured for†	
Carbohydrate	* E. coli* *	1 day	2–3 days
Glucose	100	100	100
Lactose	95	100	100
Sucrose	50	0	22
Mannitol	98	100	100
Salicin	40	0	0
Adonitol	5	0	0
Arabinose	99	92	92
Sorbitol	94	95	95
Maltose	95	100	100
Rhamnose	80	0	19
Xylose	95	97	97
Dulcitol	60	0	0
Inositol	1	0	0
Trehalose	98	30	86

*Obtained from the Manual of Clinical Microbiology, 9th Edition [69]; the prevalence (%) of positive reactions (within 2 days) is indicated.

†Proportion (%) of strains that showed a positive reaction

### Genetic backgrounds underlying delayed rhamnose and sucrose fermentation by CC119 strains

To better understand the genetic mechanisms underlying the delayed l-rhamnose and sucrose fermentation observed in the O165:H25 strains, we analysed the mutations in the *rha* and *csc* genes across the CC119ss lineage. The two phenotypes were also analysed on MacConkey agar base supplemented with l-rhamnose or sucrose using the representative CC119ss strains.

**Fig. 7.**
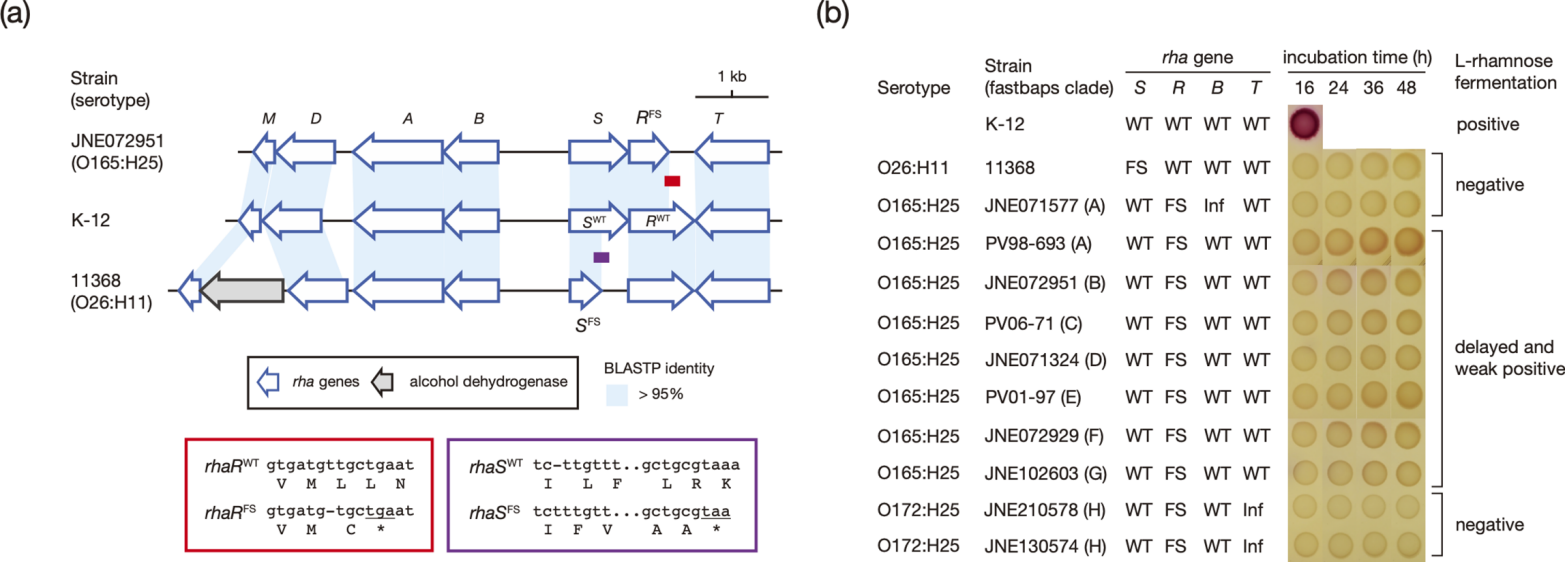
Rhamnose fermentation of CC119ss strains. (**a**) Comparison of the *rha* gene cluster in O165:H25 strain JNE072951, O26:H11 strain 11368 [4] and K-12. Homologous sequences (>95 % amino acid sequence identity) are depicted by shading. The positions and sequences of the regions containing frameshift mutations in the *rhaR* gene of strain JNE072951 and the *rhaS* gene of strain 11 368 are shown. (**b**) Rhamnose fermentation abilities of 10 CC119ss strains detected by the cell suspension spot assay. Bacterial cell suspensions (approximately 1×10^6^ c.f.u.) were spotted onto a rhamnose-containing MacConkey agar base and grown for 48 h at 37 °C. *E. coli* strain K-12 was used as a positive control, and O26:H11 strain 11 368 was used as a negative control. Along with the strain name, the intactness of the four *rha* genes in each strain is indicated (WT, wild type; FS, frameshift; Inf, in-frame insertion).

We identified five different mutations in the *rha* genes in the CC119ss strain set (Figs 7 and S10a). All strains contained a 1 bp deletion causing a frameshift in *rhaR* (referred to as *rhaR* ^FS^) (Figs 1b and 7a). The other four mutations were clade- or strain-specific: a 6 bp insertion in *rhaT* (*rhaT* ^Inf^) of clade H strains, a 3 bp insertion in *rhaB* (*rhaB* ^Inf^_1) of almost all clade A strains (91 %), a 12 bp deletion in *rhaB* (*rhaB* ^Inf^_2) of a clade D strain and a 1 bp deletion in *rhaA* (*rhaA* ^FS^) of a clade H strain (Figs 7 and S10b). All strains belonging to the two DLVs of ST119 (ST8699 and ST12464) contained intact *rha* genes (Fig. 1b and Table S1), indicating that the mutation in *rhaR* occurred in the common ancestor of CC119ss. To evaluate the effects of these mutations on the l-rhamnose fermentation ability of CC119ss strains, we selected 10 CC119ss strains representing each clade and covering various genotypes of *rha* genes and cultured them for 48 h on l-rhamnose-containing agar using *E. coli* K-12 as a positive control and *E. coli* O26:H11, which has been shown to be unable to ferment rhamnose [81], as a negative control (Fig. 7b; note that the strain carrying *rhaA* ^FS^ was not available). Seven strains containing the *rhaR* ^FS^ mutation alone showed a delayed and weak l-rhamnose fermentation phenotype, but no positive sign was observed for the three strains carrying additional mutations in *rhaB* or *rhaT* (*rhaB* ^Inf^_1 or *rhaT* ^Inf^). As RhaR is a positive regulator of the *rhaSR* operon and RhaS is required for the expression of the *rhaT* and *rhaBADM* genes [82, 83], the mutation in *rhaR* was likely responsible for the delayed and weak l-rhamnose fermentation of CC119ss strains. Although the mutations in *rhaB* and *rhaT* were in-frame mutations, they may have affected the functions of RhaB (l-rhamnulokinase) and RhaT (l-rhamnose-proton symporter), exacerbating the defects in l-rhamnose fermentation.

It should also be mentioned that a frameshift mutation in *rhaS* (1 bp insertion; *rhaS* ^FS^ in Fig. 7a) was also identified in the O26:H11 strain 11368 [4] used as a negative control. To the best of our knowledge, there are no reports on the genetic background underlying the inability of O26:H11 to ferment l-rhamnose, and this defect can be explained by the mutation in *rhaS*.

**Fig. 8.**
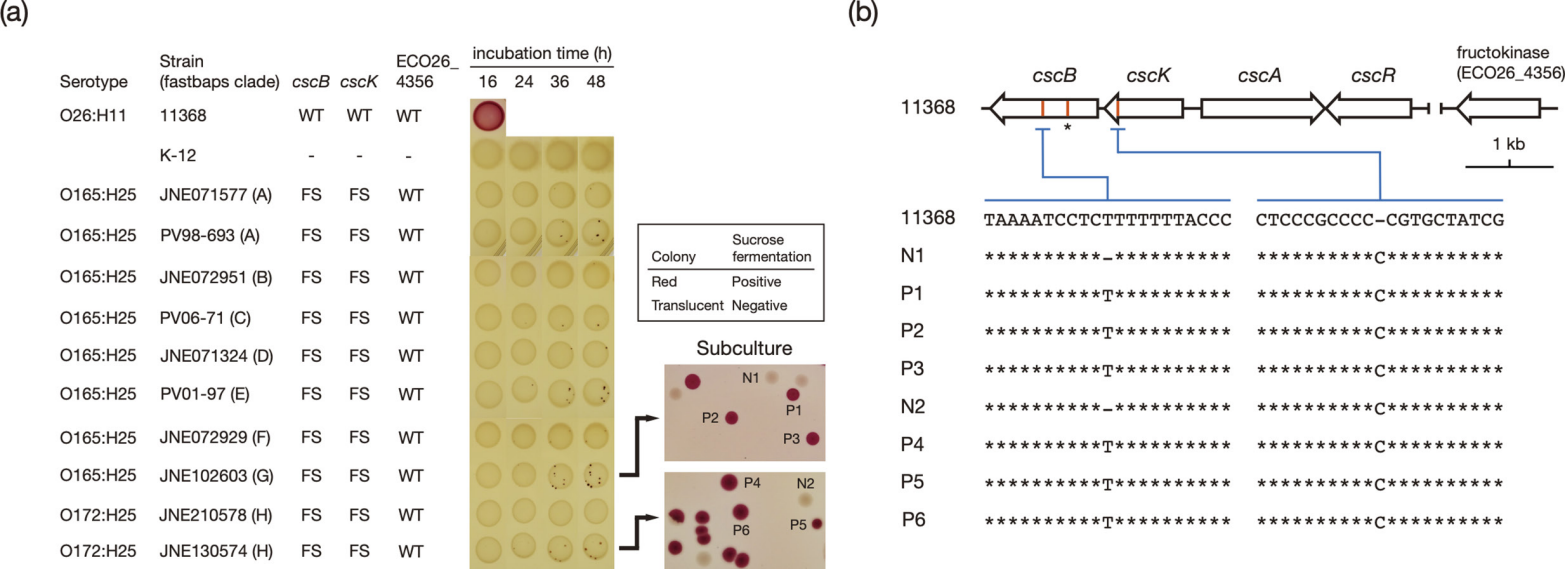
Sucrose fermentation of CC119ss strains. (**a**) The results for the spot assay on sucrose-containing MacConkey agar base are shown. The same assay as that in Fig. 7b was conducted, but O26:H11 strain 11 368 was used as a positive control and *E. coli* strain K-12 was used as a negative control. Along with the strain name, the intactness of the two *csc* genes and a fructokinase-encoding gene in each strain is indicated (WT, wild type; FS, frameshift). By picking red microcolonies in the spots of strains JNE102603 and JNE130574 and culturing them for 16 h on sucrose-containing MacConkey agar, sucrose-fermenting colonies were isolated and used in subsequent sequencing analysis. (**b**) The *csc* gene cluster and a fructokinase-encoding gene in O26:H11 strain 11368 [4] and the mutations found in CC119ss strains are shown. The positions of the insertions and deletions (InDels) in *cscB* and *cscK*, which were found in CC119ss strains, are indicated by an orange line. The sequences of the regions containing the InDels in the sucrose-fermenting colonies in (**a**) were compared with those of strain 11 368. The 1 bp deletion found in a single strain [not included in the strain set used in (**a**)] is indicated by an asterisk.

Similar analyses of the *csc* genes revealed that nearly all CC119ss strains contained frameshift mutations in *cscB* and *cscK* encoding sucrose permease and fructokinase, respectively (a 1 bp deletion in a T-tract in *cscB* and a 1 bp insertion in a C-tract in *cscK*). The only exceptions were the presence of intact *cscB* in strain JNE202009 and the lack of *cscB* in strain Z2TW04. An additional mutation in *cscB* (1 bp deletion) was found in one closed genome (H3). While all strains belonging to the DLVs of ST119 contained the same mutation in *cscK*, the *cscB* gene was intact in ST8699 and missing from ST12464 (Fig. 1b). After 24 h of cultivation of the 10 CC119ss strains analysed above (all containing the mutations in *cscB* and *cscK*) on sucrose-containing agar, we unexpectedly found small numbers of sucrose-fermenting (red) microcolonies in most strains (Fig. 8a). By subculturing the colonies of two strains (O165:H25 and O172:H25) on sucrose-containing agar, we isolated red and white colonies and sequenced the regions including the two mutations in *cscB* and *cscK*. This analysis revealed that a reverse mutation occurred in *cscB*, but not *cscK*, in all sucrose-fermenting colonies (Fig. 8b). This result indicated that the frameshift mutation in *cscB* (not that in *cscK*) was responsible for the defect in sucrose fermentation of CC119ss strains and that the delayed sucrose fermentation observed upon extended incubation in sucrose-containing Andrade’s peptone water could be explained by the emergence and proliferation of this type of revertant. It appeared that the *cscK* mutation did not affect the result of the sucrose fermentation assay in CC119ss. There are two possible explanations for this: (i) the frameshift mutation did not affect fructokinase activity, as it occurred very close to the 3′ end of *cscK,* and (ii) the presence of a homologue of this gene (showing 29 % amino acid sequence identity and 95 % coverage to ECO26_4356; Fig. 8 and Table S1) compensated for the defect in *cscK*.

## Conclusion

In this study, we determined the global population structure of CC119ss strains, including the O165:H25 and O172:H25 strains, which are understudied STEC strains due to their tellurite-sensitive phenotype. We also revealed the acquisition of the Stx2a phage, the LEE and the virulence plasmid by the common ancestor of CC119ss, identified a set of PPs/IEs highly conserved in CC119ss and showed the dynamics of Stx1a and Stx2c phages at a specific site. Importantly, we found that CC119ss strains contain major virulence genes found in O157:H7 and other major STEC serotypes, although Stx2 production levels are variable, indicating that the virulence of CC119ss strains is comparable to that of major STEC and that careful surveillance is needed. In this regard, the defects in the fermentation of four carbohydrates found in this study, particularly that of l-rhamnose, which can be fermented by most *E. coli* strains, would be useful in identifying CC119ss strains in routine laboratory examination.

## Supplementary Data

Supplementary material 1

Supplementary material 2
