# Supplementary material for: Global population structure, genomic diversity and carbohydrate fermentation characteristics of clonal complex 119 (CC119), an understudied Shiga toxin-producing E. coli (STEC) lineage including O165:H25 and O172:H25
Source: Microb Genom. 2023 Mar 23;9(3):mgen000959. doi: 10.1099/mgen.0.000959 (PMC10132054; doi:10.1099/mgen.0.000959)
Supplement: Supplementary material 1 [file mgen-9-959-s001.pdf]

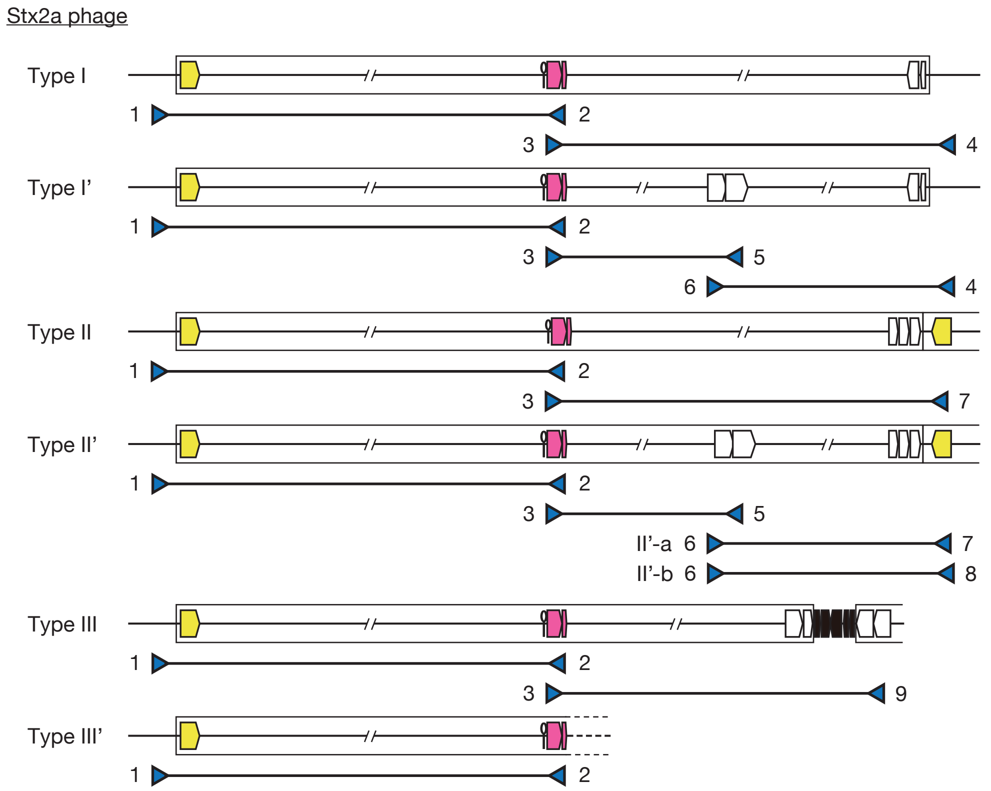

Primer	Sequence
1	GCAAGCGTCACCACAAATGC
2	TACCTTTAGCACAATCCGCCGC
3	GGAGTTTACGATAGACTTTTCGACCC
4	CGGTCCACCATTGTTGATGG
5	TCCGGTATACTTCAGCTGTGATGC
6	CTTGTTATTGACCTGAGTCTGGATGC
7	GCGACCATTTTACCGTTGAC
8	TACGCAAAACAGTTCCCCTACTCC
9	GAAGACATGACGGAGATTGAGG
10	CTTCGATTTGCTGGTGATTTCC
11	CTGGTCCAGTACTCTTTTCCG
12	GAATCAGCAATGTGCTTCCG
13	CTTCATCGAGCAGTTCCATCG
14	AACTATGGCCTCTACTGACACG
15	TCAATCATGCTGCTCACTGATCAC
16	TGGTGACAGTGAGCAACTGCA
17	TTGCGTTCTTGCCAGATGCAGA
18	GTCACCAGACAATGTAACCG
19	CGAAGGAATTTACCTTAGACTTCTCGAC
20	TCGTAATCGGCCAGTTCTTGC
21	TGAGAGCAGGTTGTGCTGTCAA
22	GCCGTTAGTGTAACTGGTGAAAGG
23	ATGGTCGCAGTAACCTTAGTGC
24	TGGTGTCCCCTGCAGGAATCG
25	GCATCAGCCACATTTGCAGCG
26	CACTGTCAGTGCTGAAGAGG
27	CTTATTGCACTGGGTGACAC

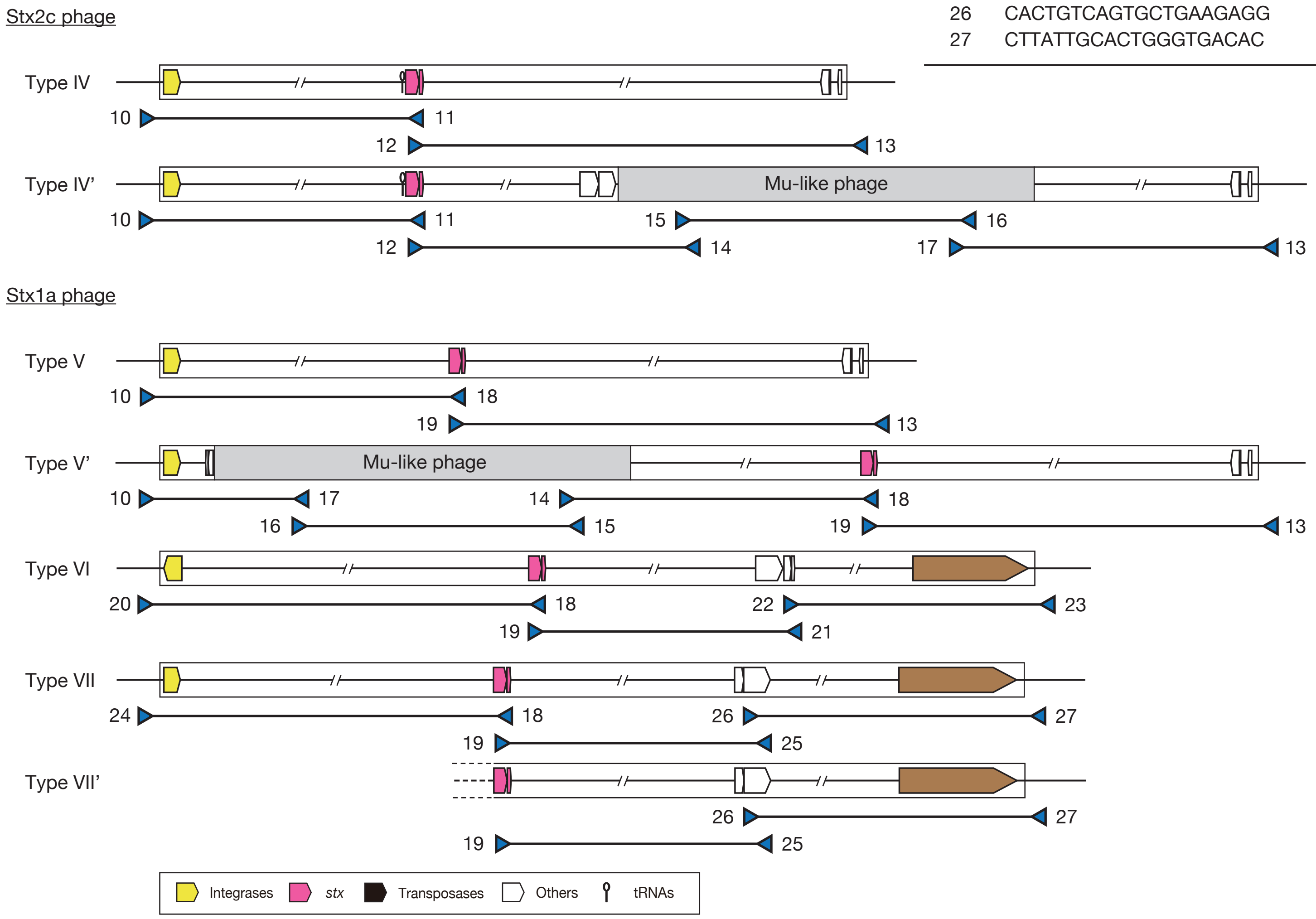

Fig. S1 Sequence determination of Stx phage genomes by seven strategies. The Stx prophage regions in each strain were divided into two, three, or four segments and amplified by long PCR as indicated. The PCR products obtained were subjected to Illumina sequencing to determine full-length prophage sequences except for type III’ and type VII’ strategies. The types of the strategies used for each Stx phage are indicated in Table S2.

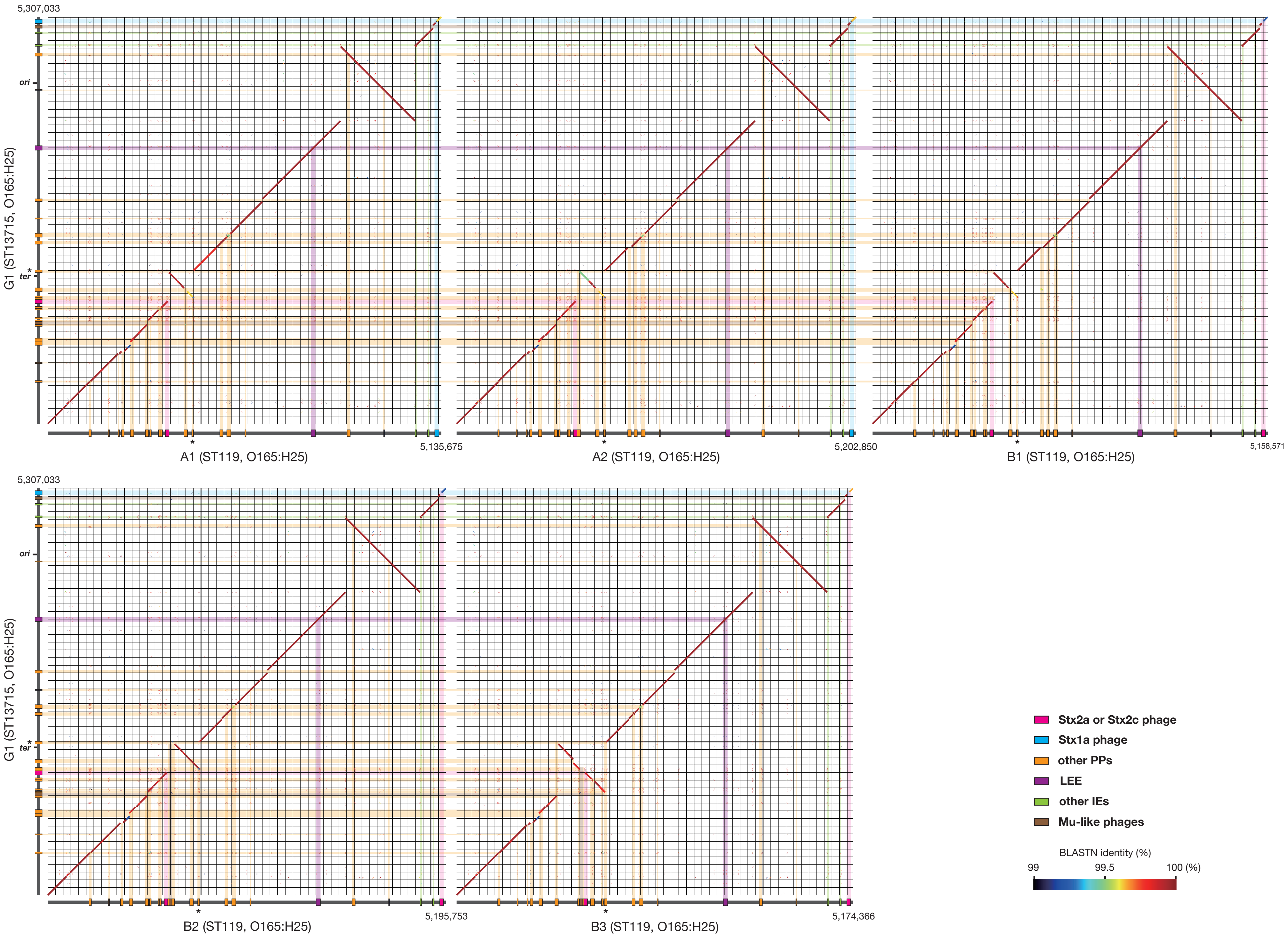

5,307,033
ori
ter
G1 (ST13715, O165:H25)
5,135,675
A1 (ST119, O165:H25)
5,202,850
A2 (ST119, O165:H25)
5,158,571
B1 (ST119, O165:H25)
5,307,033
ori
ter
G1 (ST13715, O165:H25)
5,195,753
B2 (ST119, O165:H25)
5,174,366
B3 (ST119, O165:H25)
Stx2a or Stx2c phage
Stx1a phage
other PPs
LEE
other IEs
Mu-like phages
BLASTN identity (%)
99
99.5
100 (%)

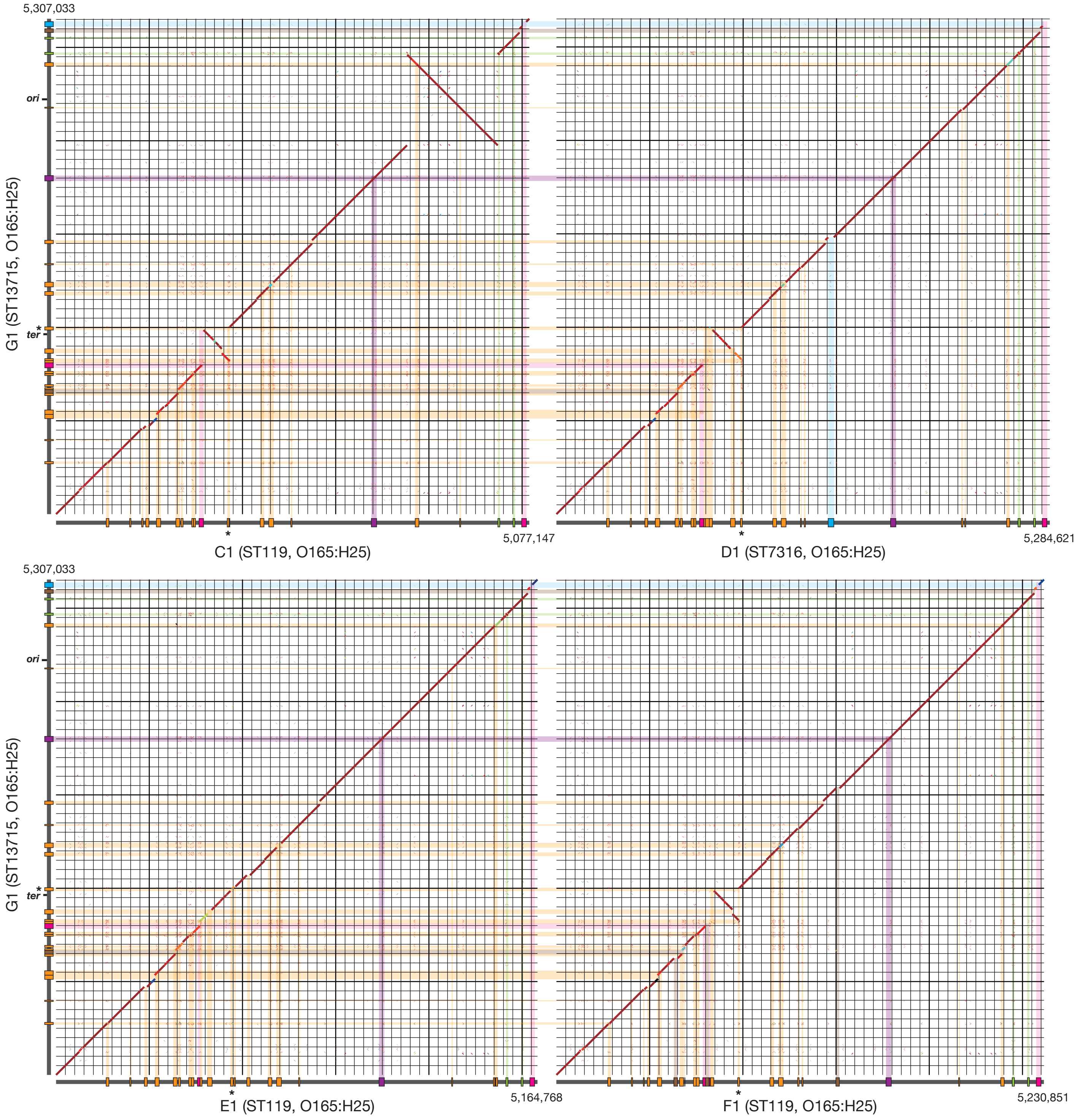

5,307,033
ori
ter
G1 (ST13715, O165:H25)
C1 (ST119, O165:H25)
5,077,147
D1 (ST7316, O165:H25)
5,284,621
5,307,033
ori
ter
G1 (ST13715, O165:H25)
E1 (ST119, O165:H25)
5,164,768
F1 (ST119, O165:H25)
5,230,851

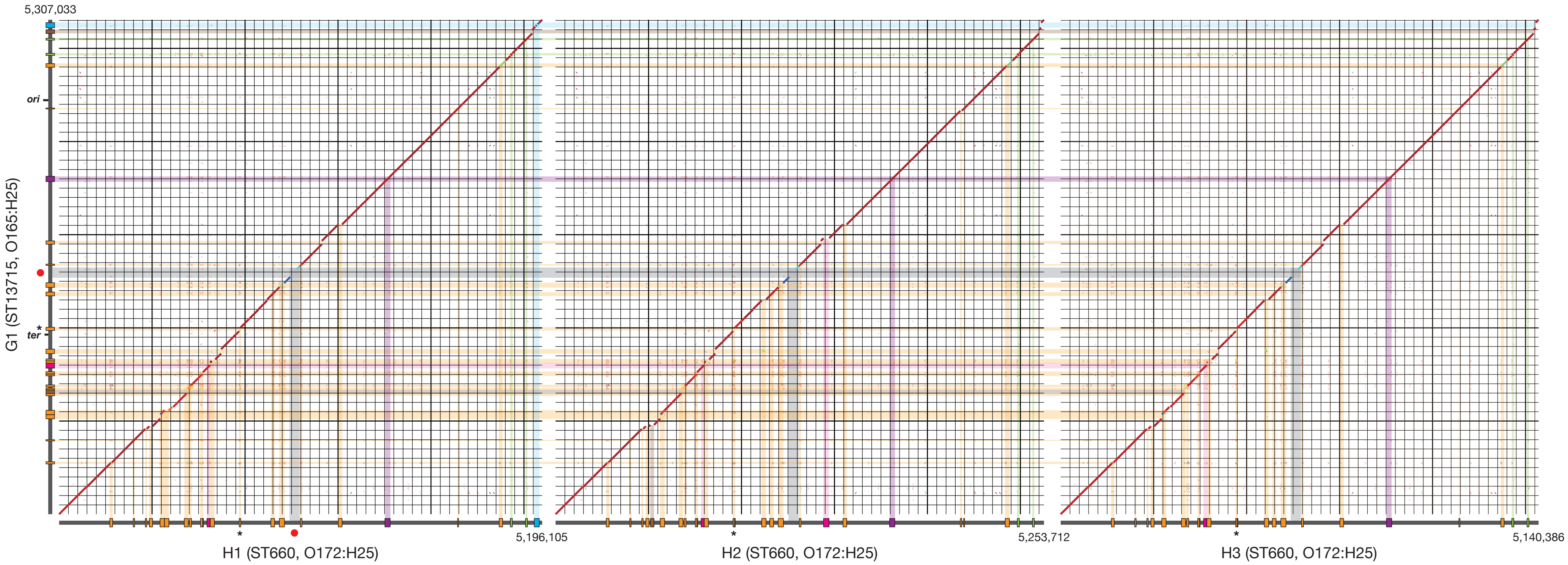

Fig. S2 Comparison of the chromosome sequences of 13 closed genomes. Dot plot matrixes of chromosome sequences between 12 closed genomes (x-axis) and that of strain G1 (y-axis) are shown. STs and serotypes of each strain are indicated in parentheses. Only the sequences with >99% identity are shown with a heat map. The positions of PPs, IEs, and Mu-like phages are indicated by boxes and shading with different colours. Grey-shaded areas are variable regions between G1 and three clade H strains. Genetic structures of this region in strains G1 and H1 (indicated by a red circle) are compared in Fig. 3 in the main text. The PPs at *ydfJ* in each strain are indicated by asterisks.

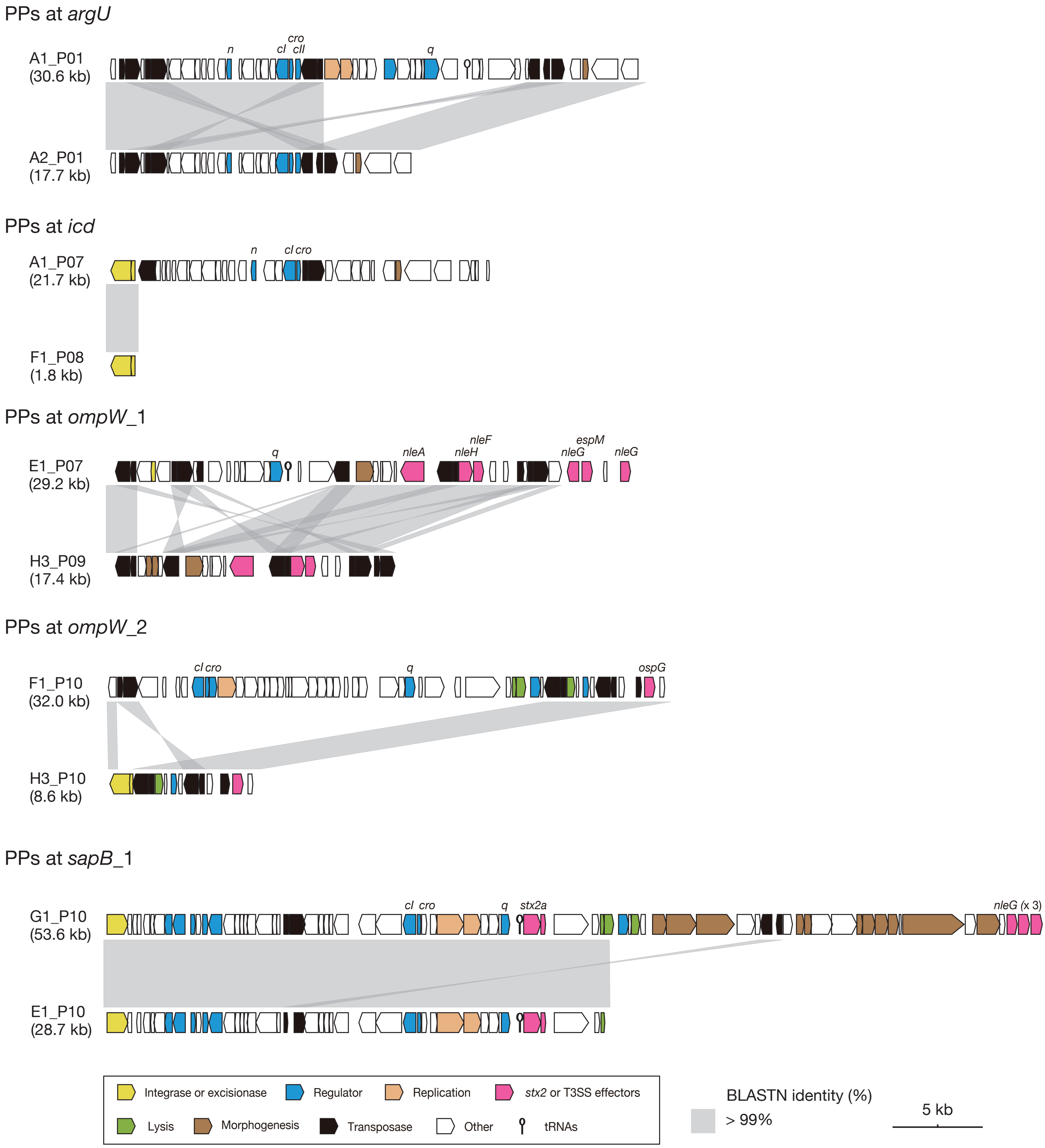

Fig. S3 Variation in the genetic structure of the PPs found at the same core PP/IE integration sites (core sites). Among the PPs found at the same site, the genetic structures of the PP pairs that exhibited the lowest alignment coverage (alignment threshold; >99% nucleotide sequence identity) are drawn to scale (see Fig. 4 and main text).

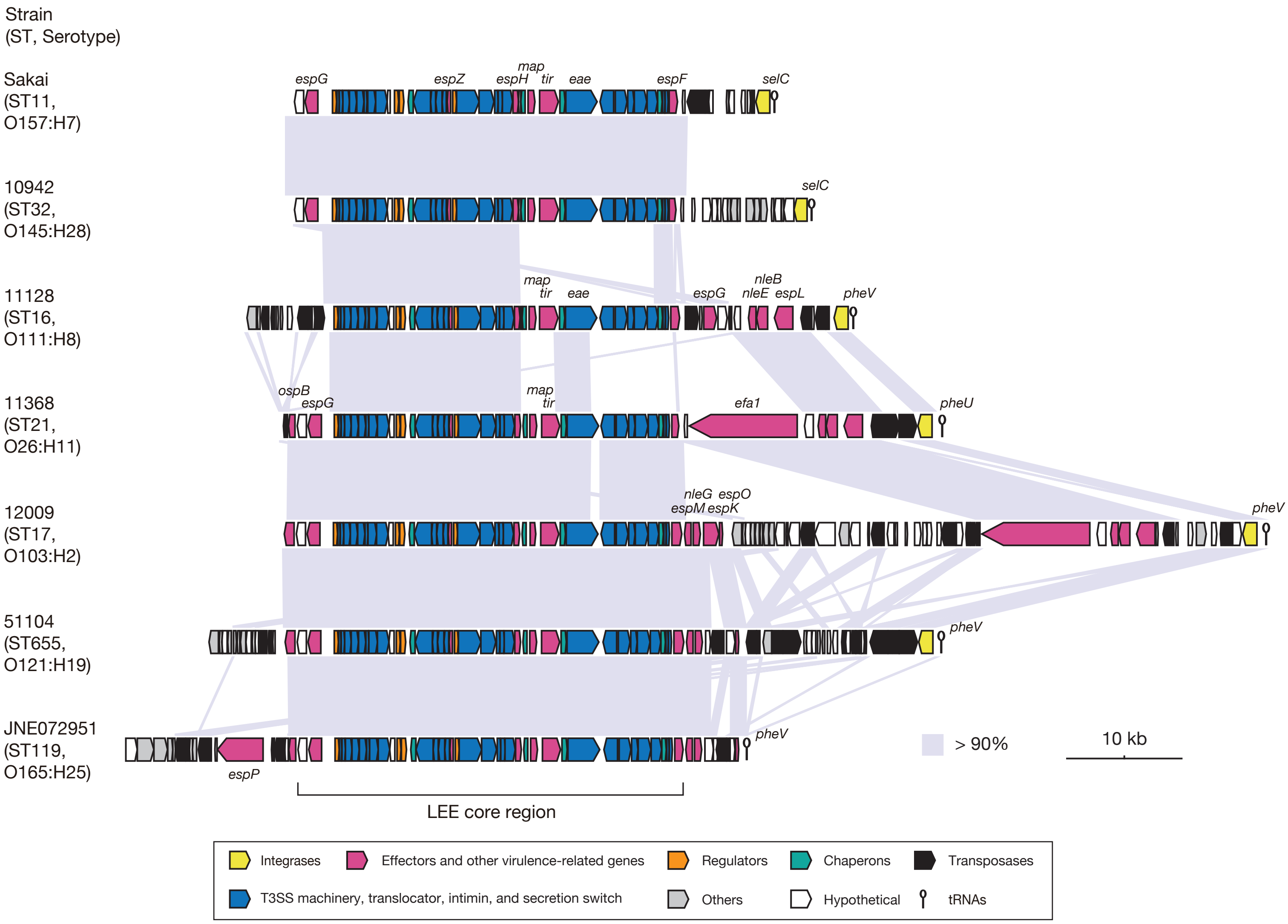

Fig. S4 The LEE element in strain JNE072951 (O165:H25) and comparison with the LEEs in STEC strains of other serotypes. The genetic structures of the LEE elements of the O165:H25 strain B1 (JNE072951) sequenced in this study and STEC strains representing the six major serotypes are drawn to scale.

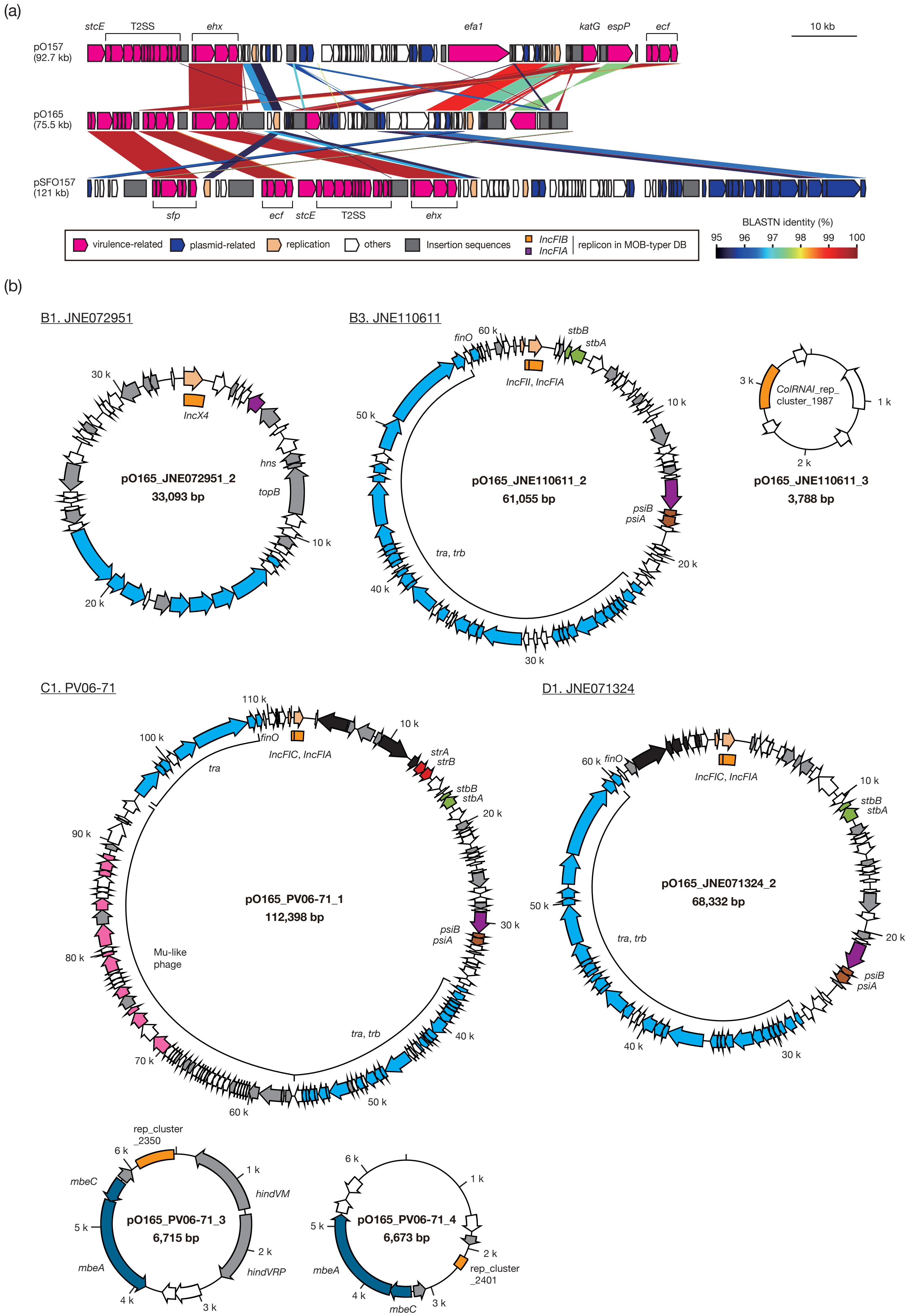
(a)
stcE
T2SS
ehx
efa1
katG
espP
ecf
10 kb
pO157
(92.7 kb)
pO165
(75.5 kb)
pSFO157
(121 kb)
sfp
ecf
stcE
T2SS
ehx
virulence-related
plasmid-related
replication
others
Insertion sequences
IncFIB
IncFIA
replicon in MOB-typer DB
BLASTN identity (%)
95
96
97
98
99
100
(b)
B1. JNE072951
30 k
IncX4
hns
topB
10 k
20 k
pO165_JNE072951_2
33,093 bp
B3. JNE110611
finO
60 k
stbB
stbA
IncFII, IncFIA
10 k
50 k
pO165_JNE110611_2
61,055 bp
psiB
psiA
tra, trb
20 k
40 k
30 k
3 k
ColRNAI_rep_
cluster_1987
1 k
2 k
pO165_JNE110611_3
3,788 bp
C1. PV06-71
110 k
10 k
100 k
finO
IncFIC, IncFIA
tra
strA
strB
stbB
stbA
20 k
90 k
pO165_PV06-71_1
112,398 bp
30 k
psiB
psiA
80 k
Mu-like
phage
tra, trb
40 k
70 k
50 k
60 k
D1. JNE071324
60 k
finO
IncFIC, IncFIA
10 k
stbB
stbA
50 k
pO165_JNE071324_2
68,332 bp
tra, trb
20 k
psiB
psiA
40 k
30 k
rep_cluster
_2350
6 k
1 k
mbeC
hindVM
5 k
pO165_PV06-71_3
6,715 bp
2 k
mbeA
hindVRP
4 k
3 k
6 k
1 k
5 k
pO165_PV06-71_4
6,673 bp
2 k
rep_cluster
_2401
mbeA
4 k
3 k
mbeC

E1. PV01-97

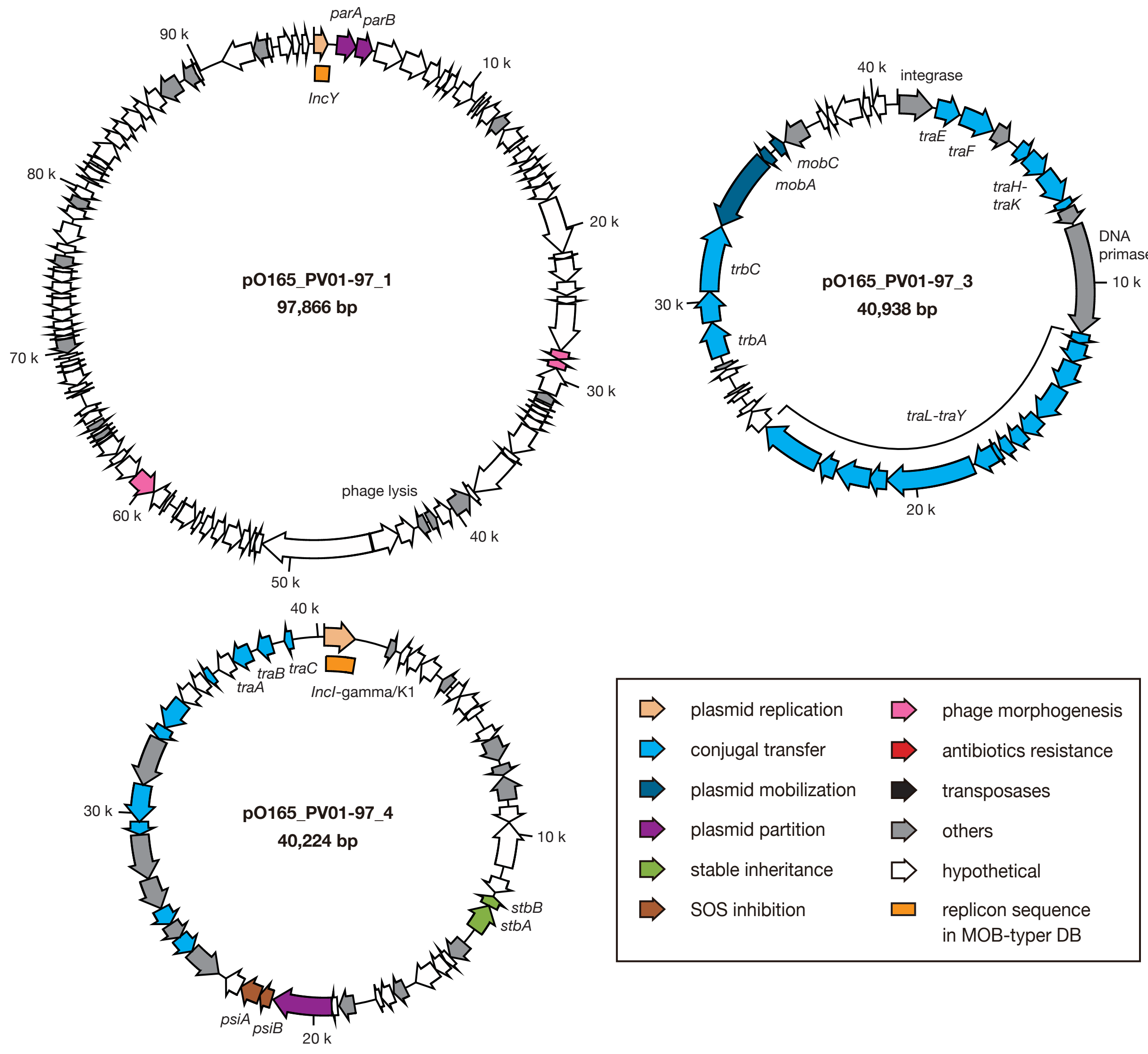

pO165_PV01-97_1
97,866 bp
parA
parB
IncY
10 k
20 k
30 k
40 k
50 k
60 k
70 k
80 k
90 k
phage lysis
pO165_PV01-97_3
40,938 bp
40 k
integrase
traE
traF
traH-
traK
DNA
primase
10 k
traL-traY
20 k
30 k
trbA
trbC
mobA
mobC
pO165_PV01-97_4
40,224 bp
40 k
traB
traC
traA
IncI-gamma/K1
10 k
stbB
stbA
20 k
psiA
psiB
30 k
plasmid replication
conjugal transfer
plasmid mobilization
plasmid partition
stable inheritance
SOS inhibition
phage morphogenesis
antibiotics resistance
transposases
others
hypothetical
replicon sequence
in MOB-typer DB

(c)

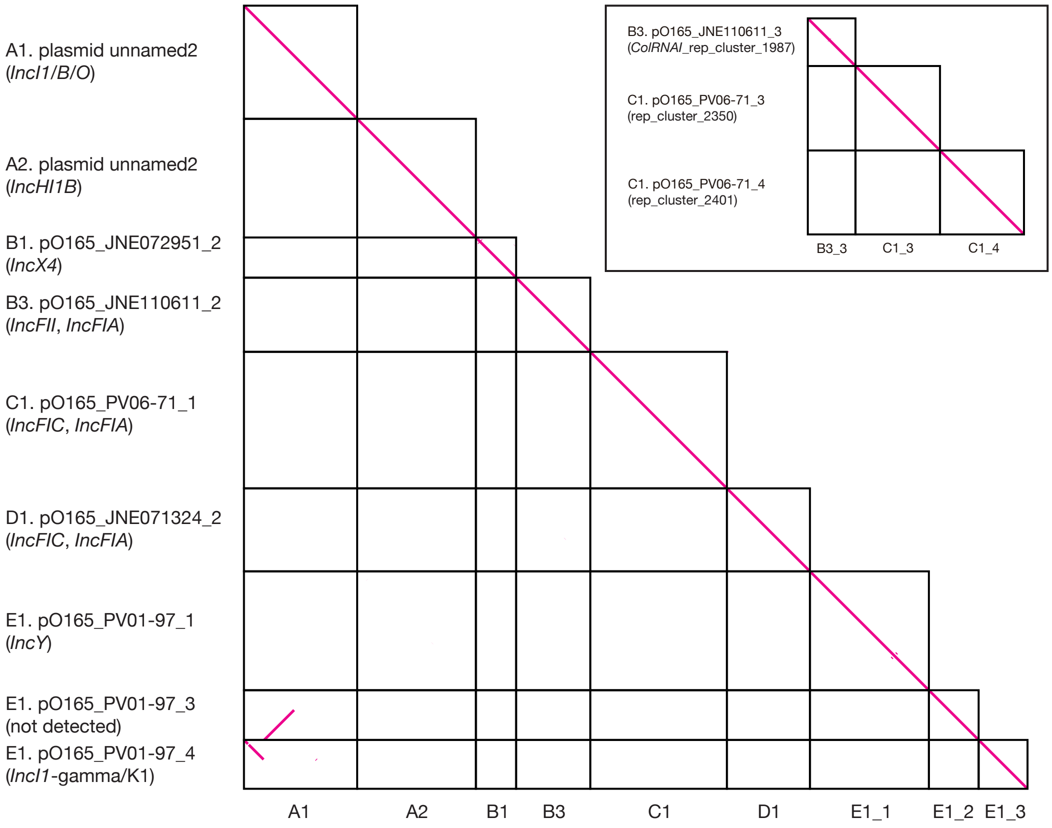

A1. plasmid unnamed2
(IncI1/B/O)
A2. plasmid unnamed2
(IncHI1B)
B1. pO165_JNE072951_2
(IncX4)
B3. pO165_JNE110611_2
(IncFII, IncFIA)
C1. pO165_PV06-71_1
(IncFIC, IncFIA)
D1. pO165_JNE071324_2
(IncFIC, IncFIA)
E1. pO165_PV01-97_1
(IncY)
E1. pO165_PV01-97_3
(not detected)
E1. pO165_PV01-97_4
(IncI1-gamma/K1)
A1
A2
B1
B3
C1
D1
E1_1
E1_2
E1_3
B3. pO165_JNE110611_3
(ColRNAI_rep_cluster_1987)
C1. pO165_PV06-71_3
(rep_cluster_2350)
C1. pO165_PV06-71_4
(rep_cluster_2401)
B3_3
C1_3
C1_4

Fig. S5 Plasmids found in the 13 closed genomes. (a) Comparison of the virulence plasmid of O165:H25 strain G1 (pO165; No. AP026762) with those of O157:H- strain 3072/96 (pSFO157; No. AF401292) and O157:H7 strain Sakai (pO157; No. AP018692). Homologous regions are depicted by coloured shading according to nucleotide sequence identity. The 'Plasmid-related' CDS include genes related to conjugation, partitioning, and SOS inhibition. (b) Circular maps of 10 nonvirulence plasmids identified in five strains sequenced in this study. (c) Dot plot analyses of the nine nonvirulence plasmids (except for three small plasmids) found in closed genomes to show their nucleotide sequence similarity (>99% identity) (unnamed plasmid 2 in strain A1, accession no. CP027327; plasmid unnamed 2 in strain A2, accession no. CP013030; pO165_JNE072951_2 in strain B1, accession no. AP026741; pO165_JNE110611_2 in strain B3, accession no. AP26744; pO165_JNE110611_3 in strain B3, accession no. AP26745; pO165_PV06-71_1 in strain C1, accession no. AP26747; pO165_PV06-71_3 in strain C1, accession no. AP26749; pO165_PV06-71_4 in strain C1, accession no. AP26750; pO165_JNE071324_2 in strain D1, accession no. AP26753; pO165_PV01-97_1 in strain E1, accession no. AP26755; pO165_PV01-97_3 in strain E1, accession no. AP26757; pO165_PV01-97_4 in strain E1, accession no. AP26758). The sequence similarity between three small plasmids (pO165_JNE110611_3, 3.8 kb; pO165_PV06-71_3, 6.7 kb; pO165_PV06-71_4, 6.7 kb) are separately analyzed, and the result is shown in the inset. Replicon types of each plasmid are indicated in parentheses.

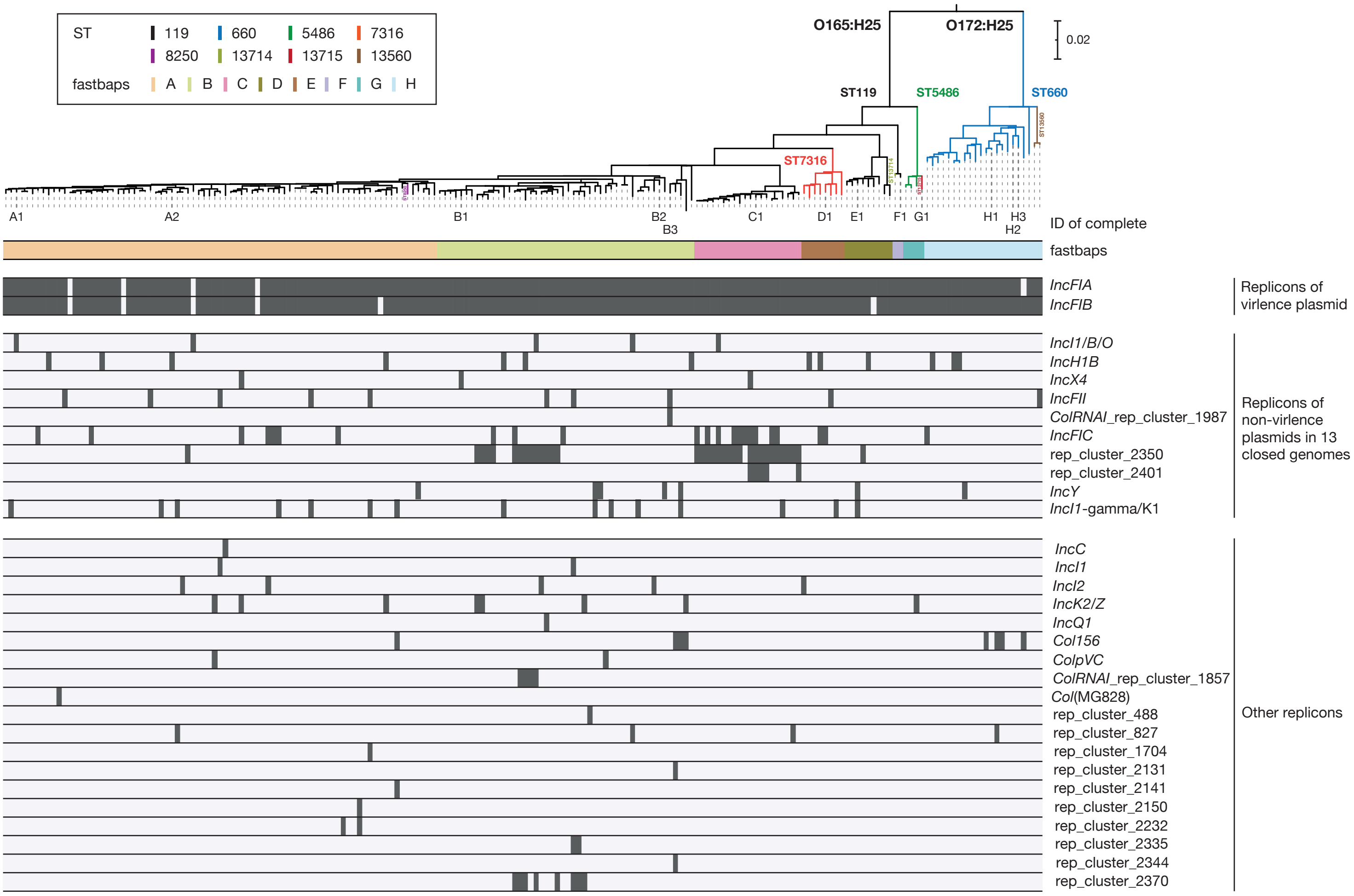

Fig. S6 Distribution of plasmid replicons in CC119ss. The results of the plasmid replicon search by MOB-typer were mapped to the ML tree of 194 CC119ss strains (the same tree as that shown in Fig. 2). The presence and absence of each replicon is indicated by a filled and open box, respectively. Bar, the mean number of nucleotide substitutions per site.

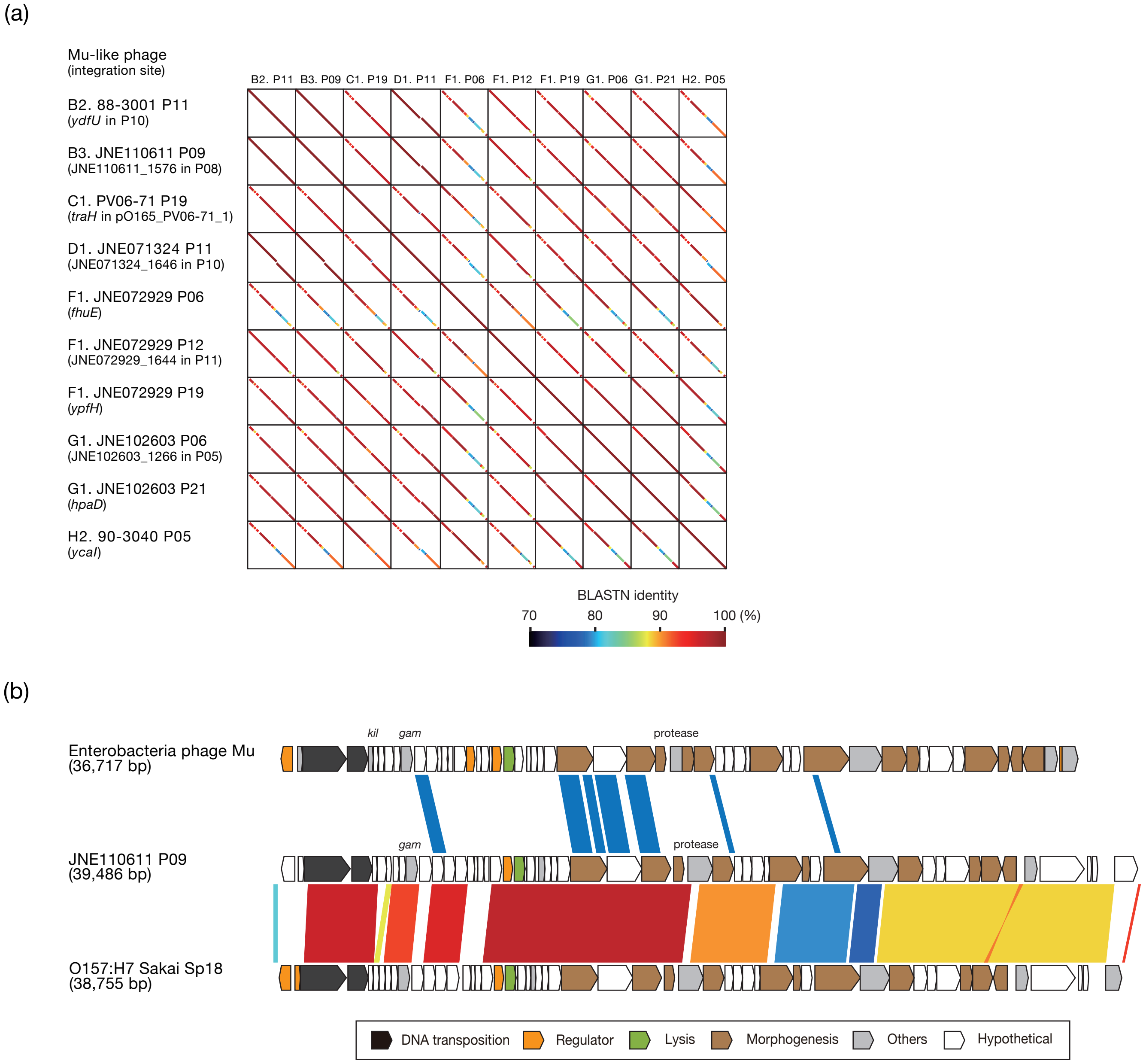

Fig. S7 Mu-like phages identified in the 13 closed genomes. (a) Dot plot presentation of the sequence similarity of the 10 Mu-like phages found in seven closed genomes. (b) Comparison of the Mu-like phage genome of O165:H25 strain B3 (JNE110611) with the enterobacteria phage Mu (No. NC_000929) and Sp18 of O157:H7 strain Sakai (No. BA000007).

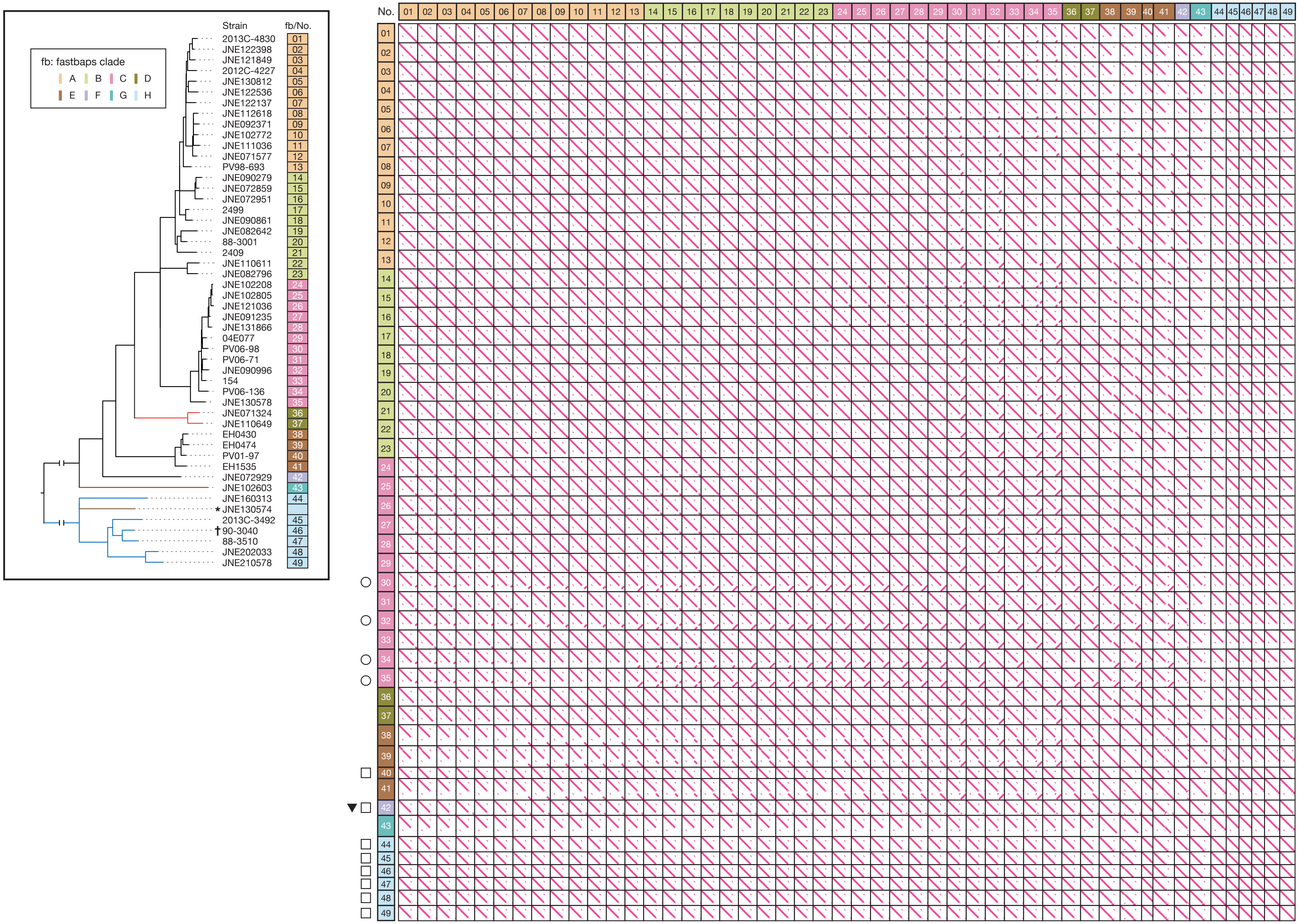
fb: fastbaps clade
A B C D
E F G H
Strain
fb/No.
2013C-4830 01
JNE122398 02
JNE121849 03
2012C-4227 04
JNE130812 05
JNE122536 06
JNE122137 07
JNE112618 08
JNE092371 09
JNE102772 10
JNE111036 11
JNE071577 12
PV98-693 13
JNE090279 14
JNE072859 15
JNE072951 16
2499 17
JNE090861 18
JNE082642 19
88-3001 20
2409 21
JNE110611 22
JNE082796 23
JNE102208 24
JNE102805 25
JNE121036 26
JNE091235 27
JNE131866 28
04E077 29
PV06-98 30
PV06-71 31
JNE090996 32
154 33
PV06-136 34
JNE130578 35
JNE071324 36
JNE110649 37
EH0430 38
EH0474 39
PV01-97 40
EH1535 41
JNE072929 42
JNE102603 43
JNE160313 44
*JNE130574
2013C-3492 45
†90-3040 46
88-3510 47
JNE202033 48
JNE210578 49
No.

Fig. S8 Conservation of Stx2a phage genome sequences between CC119ss strains. Dot plot presentation of the sequence similarity (>99% sequence identity) of Stx2a phages found at *sapB* in 49 CC119ss strains. In the inset, the phylogenetic relationship of host strains of each Stx2a phage is shown (the same ML tree of CC119ss strains is shown in Fig. 6 in the main text). Stx2a phage genomes containing an inversion in the late region and those containing a large deletion of the late region are indicated by open circles and squares, respectively. Note that the Stx2a phage genome sequence of strain JNE130574 (indicated by an asterisk) was not determined. The genetic structure of the short-tailed Stx2a phage genome of strain 90-3040 (indicted by a dagger) clearly differed from those of the long-tailed Stx2a phages at *sapB*. Therefore, these two Stx2a phage genomes were excluded from this analysis. Mu-like phage genome sequences inserted into an Stx2a phage genome (indicated by a black triangle) were removed in this analysis.

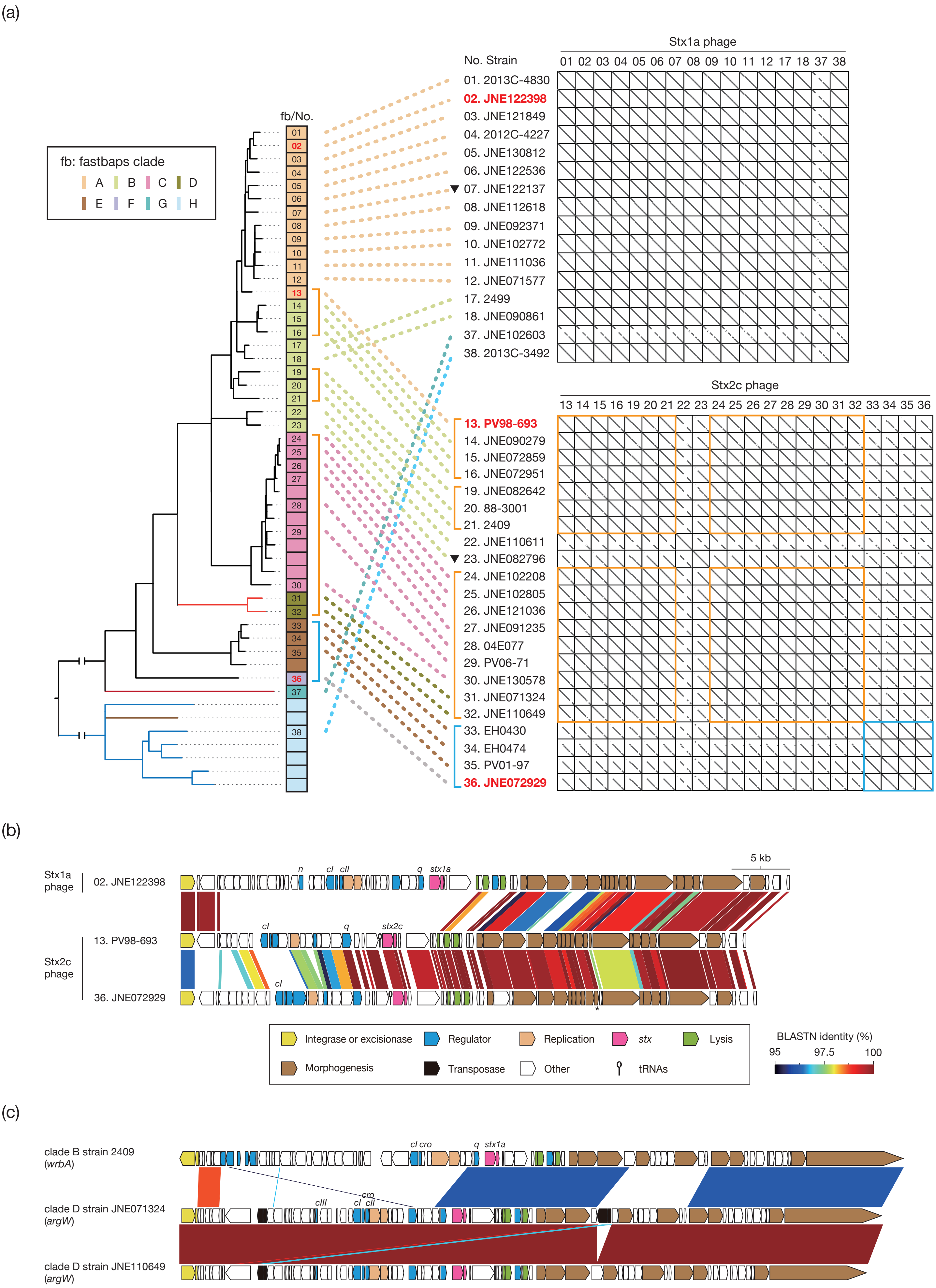
(a)
Stx1a phage
No. Strain
01 02 03 04 05 06 07 08 09 10 11 12 17 18 37 38
fb/No.
fb: fastbaps clade
A B C D
E F G H
01. 2013C-4830
02. JNE122398
03. JNE121849
04. 2012C-4227
05. JNE130812
06. JNE122536
07. JNE122137
08. JNE112618
09. JNE092371
10. JNE102772
11. JNE111036
12. JNE071577
17. 2499
18. JNE090861
37. JNE102603
38. 2013C-3492
Stx2c phage
13 14 15 16 19 20 21 22 23 24 25 26 27 28 29 30 31 32 33 34 35 36
13. PV98-693
14. JNE090279
15. JNE072859
16. JNE072951
19. JNE082642
20. 88-3001
21. 2409
22. JNE110611
23. JNE082796
24. JNE102208
25. JNE102805
26. JNE121036
27. JNE091235
28. 04E077
29. PV06-71
30. JNE130578
31. JNE071324
32. JNE110649
33. EH0430
34. EH0474
35. PV01-97
36. JNE072929
(b)
Stx1a phage
02. JNE122398
Stx2c phage
13. PV98-693
36. JNE072929
n
cI
cII
q
stx1a
stx2c
5 kb
Integrase or excisionase
Regulator
Replication
stx
Lysis
Morphogenesis
Transposase
Other
tRNAs
BLASTN identity (%)
95
97.5
100
(c)
clade B strain 2409
(wrbA)
clade D strain JNE071324
(argW)
clade D strain JNE110649
(argW)
cI cro
q
stx1a
cIII
cro
cI cII

Fig. S9 Sequence variation between Stx1a and Stx2c phages in CC119ss strains. (a) Genome sequence similarities between Stx1a and Stx2c phage genomes found at the *prfC* locus in CC119ss strains. The ML tree of host CC119ss strains (the same tree as that shown in Fig. 6 in the main text) and the dot plot presentation of sequence similarity (window size of 2 kb; >99% sequence identity) between these Stx1a and Stx2c phages are shown. Each phage is connected to its host strain by lines coloured according to the clades of host strains. Mu-like phage genome sequences inserted into Stx1a and Stx2c phage genomes (indicated by black triangles) were removed in this analysis. Stx2c phage groups with highly conserved sequences are indicated by orange or blue rectangles. (b) Sequence difference between the Stx1a and Stx2c phage genomes. Genetic structures of the Stx1a and Stx2c phages indicated by red in Panel (a) are drawn to scale. Homologous genes are depicted by coloured shading according to nucleotide sequence identity. (c) Short-tailed Stx1a phages in three O165:H25 strains. The genetic structures of these Stx1a phage genomes are drawn to scale. The integration sites of these phages are indicated in parentheses. The scale and colour patterns of genes are the same as in Panel (b), and homologous genes are also indicated in the same way as that in Panel (b).

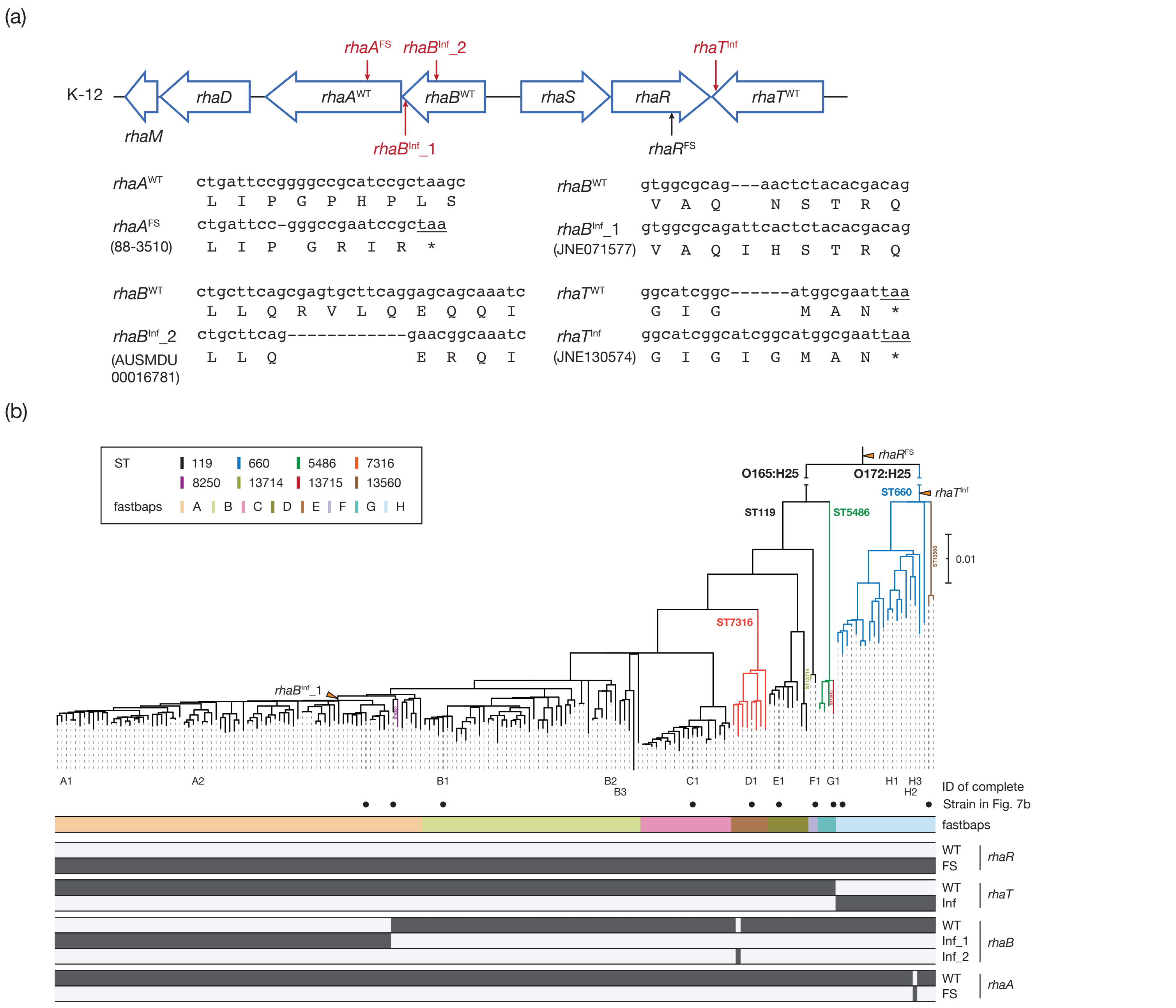

Fig. S10 The mutations in the *rha* gene identified in CC119ss strains. (a) Schematic representation of the *rha* gene cluster in K-12 and the positions of mutations identified in 194 CC119ss strains. The nucleotide sequences and deduced amino acid sequences of the frameshift and inframe mutations (indicated by red characters and arrows) are shown in the lower panel. The strain names carrying each mutation are indicated in parentheses. (b) Distribution of frameshift and in-frame mutations in the *rha* genes in CC119ss. Presence of mutations in *rhaR*, *rhaT*, *rhaB*, and *rhaA* in each strain are mapped onto the ML tree of 194 CC119ss strains (the same tree in Fig. 2 in the main text). The timing of the occurrence of three mutations (*rhaR*^{FS}, *rhaT*^{Inf}, and *rhaB*$^{Inf\_1}$) are indicated in the tree by orange triangles.
